# Comprehensive mapping of transcription terminator Rho utilization (Rut) sites across the *Bacillus subtilis* genome

**DOI:** 10.1093/nar/gkaf765

**Published:** 2025-08-13

**Authors:** Mildred Delaleau, Vladimir Bidnenko, Eric Eveno, Gergana Kostova, Johnathan C Black, Stephen McGovern, Olivier Pellegrini, Sandra Dérozier, Matthieu Jules, Ciaran Condon, Sylvain Durand, Elena Bidnenko, Marc Boudvillain

**Affiliations:** Centre de Biophysique Moléculaire, CNRS UPR4301, Affiliated with Université d’Orléans, rue Charles Sadron, 45071 Orléans cedex 2, France; Université Paris-Saclay, INRAE, AgroParisTech, Micalis Institute, 78350 Jouy-en-Josas, France; Centre de Biophysique Moléculaire, CNRS UPR4301, Affiliated with Université d’Orléans, rue Charles Sadron, 45071 Orléans cedex 2, France; Expression Génétique Microbienne (EGM), CNRS, Université Paris Cité, Institut de Biologie Physico-Chimique, 13 rue Pierre et Marie Curie, Paris 75005, France; Centre de Biophysique Moléculaire, CNRS UPR4301, Affiliated with Université d’Orléans, rue Charles Sadron, 45071 Orléans cedex 2, France; ED 549, Sciences Biologiques & Chimie du Vivant, Université d’Orléans, 45067 Orléans Cedex 2, France; Université Paris-Saclay, INRAE, AgroParisTech, Micalis Institute, 78350 Jouy-en-Josas, France; Expression Génétique Microbienne (EGM), CNRS, Université Paris Cité, Institut de Biologie Physico-Chimique, 13 rue Pierre et Marie Curie, Paris 75005, France; Université Paris-Saclay, INRAE, MaIAGE, 78350 Jouy-en-Josas, France; Université Paris-Saclay, INRAE, AgroParisTech, Micalis Institute, 78350 Jouy-en-Josas, France; Expression Génétique Microbienne (EGM), CNRS, Université Paris Cité, Institut de Biologie Physico-Chimique, 13 rue Pierre et Marie Curie, Paris 75005, France; Expression Génétique Microbienne (EGM), CNRS, Université Paris Cité, Institut de Biologie Physico-Chimique, 13 rue Pierre et Marie Curie, Paris 75005, France; Université Paris-Saclay, INRAE, AgroParisTech, Micalis Institute, 78350 Jouy-en-Josas, France; Centre de Biophysique Moléculaire, CNRS UPR4301, Affiliated with Université d’Orléans, rue Charles Sadron, 45071 Orléans cedex 2, France; ED 549, Sciences Biologiques & Chimie du Vivant, Université d’Orléans, 45067 Orléans Cedex 2, France

## Abstract

Recent evidence indicates that the bacterial Rho helicase regulates *Bacillus subtilis* gene expression in a growth-dependent manner. This regulation, along with extensive *in vivo* trimming of Rho-dependent transcript 3′-ends, complicates the identification of Rho-dependent transcription terminators using standard transcriptomic approaches. To overcome this challenge, we applied Helicase-SELEX to precisely map Rho utilization (*Rut*) sites genome-wide. Using *B. subtilis* Rho (_Bs_Rho), we identified 600 putative *Rut* sites, while the more permissive *Escherichia coli* Rho (_Ec_Rho) revealed 4189 sites, including specimens known to regulate *B. subtilis* genes. Comparative analysis showed that both enzymes recognize similar pyrimidine-rich sequences, though _Bs_Rho favors short unstructured *Rut* motifs whereas _Ec_Rho can act on presumably more structured RNAs without requiring accessory factors. *In vivo* validation of selected *Rut* sites confirmed Rho-dependent regulation and extensive PNPase-mediated processing of Rho-terminated transcripts. Collectively, our results reveal a rich and complex Rho-dependent regulatory network in *B. subtilis*, encompassing the widespread control of antisense transcription and genes/operons of both primary and secondary metabolism. Although nonessential under standard laboratory conditions, Rho thus likely contributes to *B. subtilis* fitness and survival in more demanding environments. Our comprehensive compendium of *Rut* sites offers a valuable resource for exploring this adaptive regulatory landscape.

## Graphical Abstract

**Graphical Abstract:**
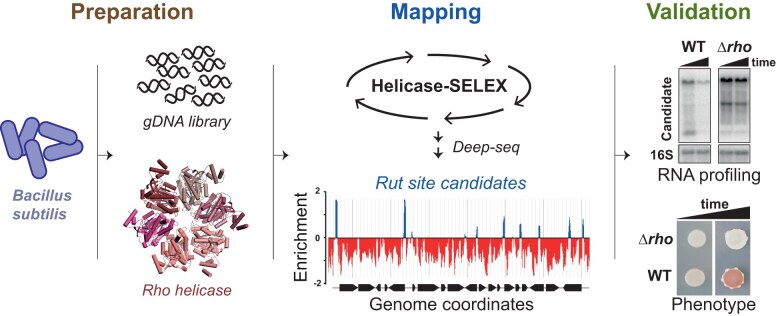

## Introduction

**Figure 1.**
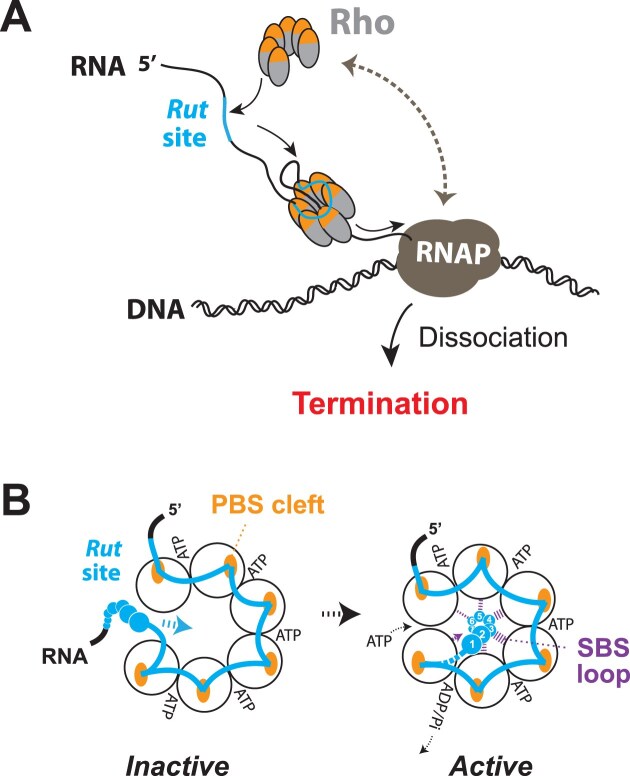
Rho-dependent termination of transcription (RDTT). (**A**) Diagram depicting the “catch-up” RDTT (aka “tethered tracking”) mechanism where Rho first binds to a *Rut* site and then translocates in an ATP-dependent manner along the nascent transcript in pursuit of the RNAP [4, 5]. The brown double arrow illustrates an alternative “stand-by” RDTT pathway where Rho is pre-bound to RNAP [7, 8]. (**B**) Diagram depicting how RNA binding at the level of a *Rut* site activates the Rho enzyme. The corkscrewed configuration of the open Rho hexamer facilitates entry of the RNA chain bound to the PBS (left). The downstream part of the *Rut* site can then contact the SBS loops to trigger the closure and catalytic activation of the Rho hexamer (right).

Bacteria are remarkably diverse and adaptable organisms that have evolved sophisticated regulatory mechanisms to fine-tune gene expression, enabling them to colonize and thrive in a wide range of ecological niches. Among these regulatory mechanisms, transcription termination has received growing attention because it can both shape the bacterial transcriptome at global scale and dynamically regulate individual genes in response to environmental cues [1, 2]. Bacteria use two main mechanisms for transcription termination [3]. On one hand, intrinsic (Rho-independent) termination (IT) relies on the presence of specific motifs in the nascent transcript—composed of a hairpin structure followed by a run of uracil residues—that can destabilize the transcription elongation complex (TEC). On the other hand, Rho-dependent transcription termination (RDTT) requires the action of the ring-shaped RNA helicase Rho. A key step in the RDTT pathway is the recognition of a Rho utilization (*Rut*) site in the nascent transcript by the Rho helicase, which then uses its ATPase-fueled 5′-to-3′ RNA translocase activity to catch up with RNA polymerase (RNAP) and dissociate the TEC [4, 5] (Fig. 1A). Rho can also first bind RNAP and then scan the emerging RNA continuously from this RNAP-bound position, triggering RDTT when it detects a *Rut* site [6–8].

Despite the key role of the *Rut* sites in prompting RDTT, they cannot be identified reliably from sequence analysis alone. This is largely due to the complex RNA binding trajectory on the Rho hexamer (Fig. 1B) where some of the RNA contacts are likely redundant and adjustable or dispensable. Indeed, studies of the Rho factor from *Escherichia coli* (_Ec_Rho) support that not all of the six RNA contacts to the crown-like Primary Binding Site (PBS) of Rho (Fig. 1B) are systematically required for the formation of a stable or catalytically productive interaction [9, 10]. In addition, while PBS clefts on each _Ec_Rho subunit preferentially accommodate YC-rich motifs (where Y is a C or U residue) [11], the RNA segments linking PBS clefts from adjacent subunits can vary in length and sequence [4, 12]. Consequently, only loose consensus rules have to date been determined for *Rut* sites (and by extension for RDTT terminators) in *E. coli*: they tend to have YC-rich sequences with a U/C > G-bias and have a lower propensity than *Rut*-less sequences to form stable RNA secondary structures [4, 10, 13, 14]. Whether similar or stricter rules apply to RDTT in other species has not yet been determined.

A long-standing view is that RDTT plays a more critical role in Gram-negative bacteria such as *E. coli* than in Gram-positive species such as *Bacillus subtilis*. This perception arose from observations that most Gram-positive species are resistant to the Rho inhibitor bicyclomycin [15] and can still grow in standard laboratory conditions after disruption of the *rho* gene [16, 17]. Furthermore, computational predictions of IT terminators support IT as the dominant mechanism in Gram-positive Firmicutes [18]. However, comparative transcriptome profiling of wild-type (WT) and Δ*rho* strains of *Staphylococcus aureus* [19, 20] and *B. subtilis* [21–23] now provides a more nuanced perspective on the importance of RDTT in these species. Indeed, *rho* deletion has genome-wide effects in both species, notably causing a marked increase of pervasive, mostly antisense transcription. While not lethal under standard growth conditions, this widespread dysregulation of gene expression impacts bacterial fitness, enhancing *S. aureus* virulence [20] and disrupting cell differentiation in *B. subtilis* [22]. Several analyses also revealed that, despite *rho* autoregulation [16], cellular Rho levels are not constant in *B. subtilis* but vary with the growth phase or the cellular compartment during sporulation, thereby globally adjusting RDTT to specific growth or cell differentiation needs [21, 23–25]. Furthermore, transcriptome profiling and Term-seq analyses revealed that RDTT and IT are not strictly independent in *B. subtilis*, with Rho enhancing termination at ∼10% of the IT terminators that are active during exponential growth [21, 26]. This subset of IT signals is characterized by weaker hairpins and U-rich tracts, and two mechanisms appear to explain stimulation by Rho: (i) Rho binds the nascent transcript upstream, preventing the formation of “anti-terminator” RNA structures that would outcompete the weaker IT hairpin, or (ii) Rho triggers RDTT downstream when RNAP reads through the weak IT signal [26].

These discoveries highlight the complexity of the gene expression programs of *B. subtilis* but also emphasize the difficulty to map RDTT sites comprehensively using standard transcriptome profiling or Term-seq approaches, as the RDTT landscape likely shifts depending on growth phase or culture conditions. Mapping RDTT sites is further complicated by the activity of 3′-to-5′ exoribonucleases, which can extensively degrade the 3′-ends of Rho-terminated transcripts, as demonstrated in *E. coli* [27, 28]. This trimming process can span hundreds of nucleotides (nt) and halts only when a sufficiently stable RNA structure sterically blocks exoribonuclease activity. Consequently, the *in vivo* 3′-ends of Rho-terminated transcripts often do not coincide with the actual termination sites [27, 28], making precise RDTT site mapping significantly more challenging.

To address these mapping challenges, we developed an alternative and complementary *in vitro* methodology, Helicase-SELEX (hereafter H-SELEX), which leverages the RNA helicase activity of Rho to identify genuine *Rut*-like sequences within large genomic libraries [10]. H-SELEX precisely mapped thousands of putative *Rut* sites across the *E. coli* genome [10] and prompted the discovery of RDTT’s primary role in regulating cold shock genes in *E. coli* and *Salmonella* [29]. Using H-SELEX, we now identify thousands of sequences encoding putative *Rut* sites across the *B. subtilis* genome. We show that these *Rut* sequences are preferentially positioned antisense to genes, consistent with the primordial role of Rho in silencing pervasive antisense transcription in *B. subtilis* [21, 22]. We highlight specific cases where *Rut* sequences mediate gene regulation in the sense orientation or contribute to complex regulatory cascades. Finally, we demonstrate that PNPase extensively degrades the 3′-ends of Rho-terminated transcripts in *B. subtilis*. Altogether, our compendium of putative *Rut* sites provides a unique resource to explore the full RDTT landscape of *B. subtilis*, independent of mitigating factors such as post-transcriptional RNA processing, growth stage, or culture conditions.

## Materials and methods

### Reagents

Unless specified otherwise, chemicals and enzymes were obtained from Sigma–Aldrich and New England Biolabs, respectively. Radionucleotides were purchased from Hartmann Analytics GmbH and synthetic oligonucleotides (listed in Supplementary Table S1) were supplied by Eurogentec.

### Biological resources

All *B. subtilis* strains used in this study (listed in Supplementary Table S2 alongside plasmids) are derivatives of *B. subtilis* 168 *trp^+^* BsB1 [21]. The *E. coli* strain TG1 (Stratagene) was used for construction of plasmids. Expression plasmid pET28a-BsRho was constructed by cloning the *B. subtilis rho* gene between the NdeI and SalI sites of the pET28a vector (Novagen), maintaining the correct reading frame with the vector’s N-terminal His_6_ tag.

### Preparation of proteins

Rho protein concentrations are expressed in hexamers throughout the manuscript. The _Ec_Rho protein was prepared as described previously [30]. The _Bs_Rho protein was overexpressed in *E. coli* Rosetta-II cells (Novagen) harboring the pET28a-BsRho plasmid. Cells were pelleted and resuspended in Lysis buffer (20 mM Tris–HCl pH 9, 50 mM Na_2_HPO_4_, 300 mM NaCl, 10% glycerol, and 0.1% Triton X-100) supplemented with cOmplet protease inhibitor cocktail (Roche). Lysozyme (0.5 mg/ml) was added and the mixture was gently stirred for 15 min at room temperature before addition of MgCl_2_ (24 mM) and DNase I (4 μg/ml) and further incubation for 10 min at room temperature. The lysate was then sonicated on ice using a Bioblock Vibra-Cell 75 115 apparatus (40% amplitude, 5 min total: 30s on/ 30s off cycles). Cell debris were removed by centrifugation at 17 000 *× g* for 20 min at 4°C. The clarified supernatant was supplemented with imidazole (1 mM final concentration) and sequentially filtered through 0.5 and 0.2 μm filters. Protein purification was performed at room temperature by affinity chromatography using a 5 ml Ni-NTA His-Trap column (Cytiva) pre-equilibrated with buffer A (20 mM Tris–HCl pH 9, 300 mM NaCl, 5% glycerol, 50 mM Na_2_HPO_4_, and 5 mM imidazole). The crude lysate was loaded onto the column, which was washed sequentially with 20 ml of buffer A and 20 ml of buffer B (20 mM Tris–HCl pH 9, 600 mM NaCl, 5% glycerol, 50 mM Na_2_HPO_4_, and 20 mM imidazole). The _Bs_Rho protein was eluted with 30 ml of elution buffer (20 mM Tris–HCl pH 9, 300 mM NaCl, 5% glycerol, 50 mM Na_2_HPO_4_, and 250 mM imidazole). A white precipitate formed in the eluted fractions suggesting that _Bs_Rho was near its solubility limit. The precipitate was eliminated by centrifugation and the supernatant dialyzed overnight at room temperature in a 3500 MWCO dialysis membrane (Spectrum labs) against dialysis buffer (20 mM Tris–HCl pH 9, 50 mM Na_2_HPO_4_, 150 mM NaCl, and 10% glycerol). The dialyzed _Bs_Rho was filtered through a 0.2 μm filter, adjusted to a final glycerol concentration of 40% (v/v), and stored as 1.4 μM aliquots at −80°C.

### Preparation of the B. subtilis gDNA fragment library

The library of DNA fragments was prepared as described previously [10] using genomic DNA (gDNA) from *B. subtilis* BsB1 strain. Briefly, gDNA was dissolved in TE buffer (10 mM Tris–HCl, pH 8, 1 mM EDTA) and incubated with 50 μg/ml RNase A for 1 h at 37°C, before sonication on ice with a Vibracell 75115 apparatus equipped with a microprobe (20% amplitude; 30 cycles of 30s on/ 30s off), phenol extraction, and ethanol precipitation. The fragmented gDNA was dissolved in TE buffer and its concentration determined with the Quant-iT kit on a Nanodrop 3300 spectrofluorimeter (Thermo-Fisher Scientific). Then, 25 μg of fragmented gDNA were mixed with 1.9 nmole of primer R45-ran-rev in Klenow buffer (50 mM NaCl, 10 mM Tris–HCl, pH 7.9, 10 mM MgCl_2_, and 1 mM dithiothreitol (DTT)), incubated at 95°C for 5 min, and then chilled on ice before addition of dNTPs (0.3 mM each, final concentration) and 0.5U/μl of Klenow polymerase. The mixture was incubated at 4°C for 5 min, 25°C for 25 min, and 50°C for 5 min before addition of 10 mM EDTA and further incubation for 10 min at 75°C. First-strand DNA products were purified on a GeneJET column (Thermo-Fisher Scientific), mixed with 3.8 nmol of ARN107-ran-For primer in Klenow buffer, and extended with 0.5 U/μl of Klenow polymerase following the same incubation and column purification steps described above. The gDNA fragments in the desired size range were purified by 7% denaturing polyacrylamide gel electrophoresis (PAGE) [31]. The resulting gDNA fragment library (∼175 ng) was amplified by standard polymerase chain reaction (PCR; 13 cycles) with Vent polymerase using primers FWD and REV to introduce the sequences of the T7 promoter and reporter pairing region. The library was purified on GeneJET columns, quantified with the Quant-iT kit, and stored in TE buffer at −20°C before use.

### H-SELEX enrichment experiments

Each round of H-SELEX was performed on a customized TECAN Evo150 liquid handling platform equipped with 4-channel Air LiHa and RoMa gripper arms, Te-Vacs filtration module, ALPAQUA 96-well magnet, and INHECO modules for plate cooling, heating, shacking, and thermo-cycling. Four samples were handled in parallel in 96-well Deepwell protein lobind plates (Eppendorf) by the TECAN robot (two replicates for each enrichment with _Bs_Rho or _Ec_Rho, all from the same starting gDNA fragment library, R_0_). The following protocol is for a single sample/channel. Around 64 pmoles of the gDNA fragment library (round 1) or ∼32 pmoles of the DNA library from the previous round were transcribed with T7 RNAP for 2 h at 37°C. Then, half of the crude transcription reaction was mixed with 240 pmoles of biotinylated oligonucleotide SEL and 480 pmoles of oligonucleotide BLOCK. The mixture was heated for 2 min at 90°C and then cooled to 37°C. Meanwhile, 200 μl of magnetic streptavidin bead slurry (Invitrogen Dynabead T1) were washed twice with BW buffer (1 M KCl, 5 mM Tris–Cl, pH 7.5, 0.5 mM EDTA) and once with helicase buffer (150 mM Potassium Acetate, 100 nM CaCl_2_, 10 mM NaCl, and 25 mM EPPS pH 9.0). Then, the sample mixture was added to the beads and incubated under shaking (300 rpm) for 1 h at 22°C. Beads were separated from supernatant on the ALPAQUA magnet and then washed thoroughly, once with BW buffer, once with 1× Denhardt’s solution (Thermo-Fisher Scientific), and twice with helicase buffer. The bead-affixed duplexes were pre-incubated under shaking (300 rpm) with 80 nM Rho in helicase buffer for 5 min at 37°C before addition of 1 mM adenosine diphosphate (ADP) and 400 nM oligonucleotide TRAP, and further incubation under shaking (300 rpm) for 15 min at 37°C. The beads were placed on the ALPAQUA magnet and the supernatant discarded to eliminate potential non-ATP-dependent side-reaction products. The remaining bead-affixed duplexes were washed with 1× Denhardt’s solution and then with helicase buffer, before pre-incubation under shaking (300 rpm) in helicase buffer with 80 nM Rho for 5 min at 37°C. Then, 1 mM adenosine triphosphate (ATP) and 400 nM oligonucleotide TRAP were added and the mixture was incubated under shaking (300 rpm) at 37°C for the round-dependent duration indicated in Supplementary Table S3. The helicase reaction was quenched with 20 mM EDTA and the supernatant containing the released RNA strands was magnetically separated from the beads and loaded on a RNA clean&concentrator column (Zymo Research). The RNA strands were purified and eluted from the column, following manufacturer’s instructions. The eluate was mixed with dNTPs (0.5 mM each) and 50 pmoles of REV primer in Maxima reverse transcription buffer (Thermo-Fisher Scientific) adjusted to 1×. The mixture was incubated for 5 min at 70°C before addition of 400 U of Maxima H-minus reverse transcriptase (Thermo-Fisher Scientific) and further incubation for 1 h at 50°C and 15 min at 70°C. The single-stranded DNA (ssDNA) products were amplified by PCR (12 cycles) with 50 U of Taq DNA polymerase using FWD and REV primers (0.5 μM, final concentrations). The resulting double-stranded DNA (dsDNA) template library was purified on GeneJET columns before use in the next automated H-SELEX round. The DNA concentration in the library was determined with the Quant-iT kit.

### Sequence processing and bioinformatics analysis

**Figure 2.**
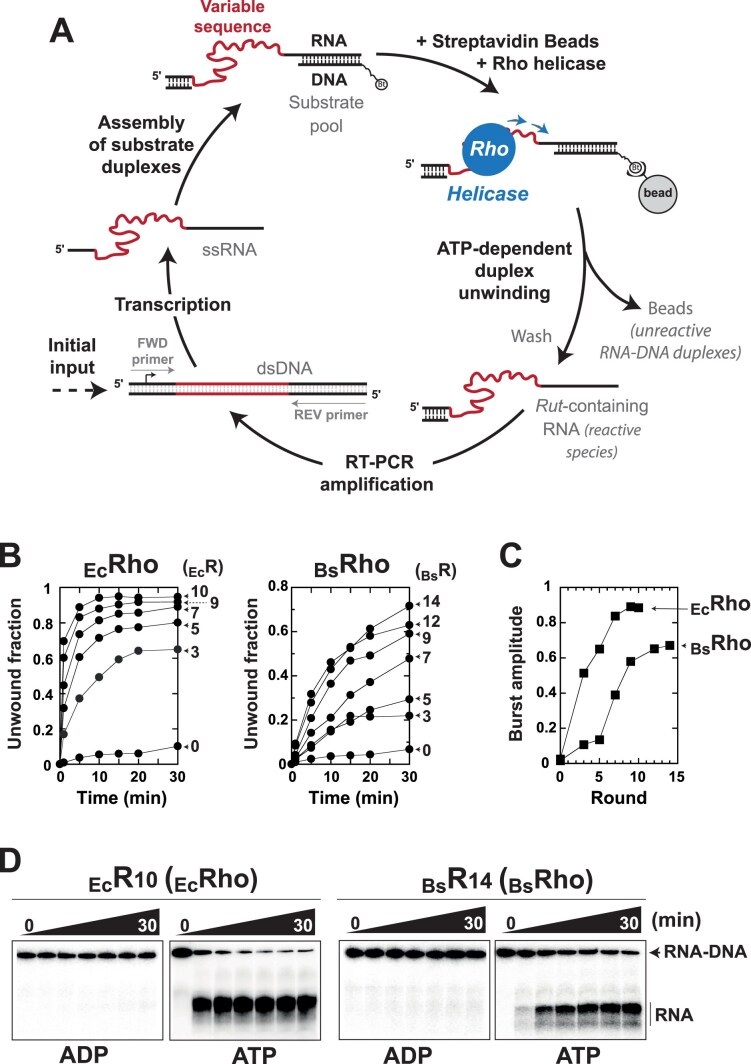
Mapping putative *Rut* sites in the genome of *B. subtilis 168* using H-SELEX. (**A**) Schematic representation of the main steps in an H-SELEX round. (**B**) Progressive H-SELEX enrichment of active RNA–DNA duplexes, as shown by the kinetics of library duplex unwinding by _Ec_Rho (left) or _Bs_Rho (right) across H-SELEX rounds. The library duplicates _Ec_R_(n)a,b_ (left) or _Bs_R_(n)a,b_ (right) were mixed at a 1:1 molar ratio for analysis. (**C**) Burst amplitudes derived from fitting panel (B) data to a pseudo-first order kinetics equation [34]. Maximal enrichment, inferred from plateauing amplitude, occurs at rounds 10 and 14 for _Ec_Rho and _Bs_Rho, respectively. Experiments were not duplicated to limit depletion of the libraries before deep sequencing [10]. (**D**) Representative PAGE gels showing the ATP-dependent activity of the _Ec_Rho and _Bs_Rho helicases on the final H-SELEX duplex libraries.

Bioinformatics analyses were performed using in-house Python scripts and publicly available software tools (https://usegalaxy.eu/), following previously described procedures [32] with minor modifications. Briefly, the dsDNA libraries obtained by H-SELEX were analyzed by 2 × 150 base paired-end sequencing on a NextSeq Illumina instrument at the I2BC sequencing facility (CNRS, Gif-sur-Yvette, France). Starting, blunt-ended dsDNA pools (∼2.5 μg each) were processed by the I2BC facility using standard procedures and the NextSeq2000 P2 (300-cycles) reagents kit (Illumina). Samples were supplemented with coliphage phiX174 DNA to prevent instrumental artefacts due to the invariant end sequences of the H-SELEX duplexes (Fig. 2A). Multiplexed sequencing of the pools resulted in ∼25 × 10^6^ to ∼36 × 10^6^ paired-end reads per pool after quality control filtering (FastQC v0.74) and adapter trimming (Cutadapt3.2). Paired-end reads (≥30 nt) were concatenated and coliphage phiX174 sequences were expunged from sequence libraries by selecting reads containing FWD and REV primer-binding regions using Fastaq-join v2.0.1 (Max % difference between matching segments: 8; Min length of matching segments: 6), Filter Fasta v2.1, and Cutadapt v4.9 tools. Curated reads were mapped on the *B. subtilis* 168 genome (NC_000964.3) with Bowtie2 (v2.4.2, default options). Coverage and RPM normalization were performed with Bedtools v2.31. A representative snapshot of normalized read coverages for the starting R_0_ library and for the enriched _Bs_R_14a,b_ and _Ec_R_10,a,b_ library replicates is provided in Supplementary Fig. S1A. To obtain the mean log_10_FE profiles shown in the figures, the normalized coverage (BEDGRAPH) files for the enriched sample replicates were first merged with Bedtools and then averaged with a dedicated Python script. Then, the mean log_10_FE profiles were computed with MACS2 bdgcmp v2.2.9 using the averaged _Bs_R_14_ or _Ec_R_10_ sample file as input and the R_0_ sample file as control. Peak calling was performed with MACS2 bdgpeakcall v2.2.9 (cutoff: 0; Min peak length: 30; Max gap: 30; median R_0_ read count per nt position ≥ 1; median log_10_FE ≥ 0.3) or PEAKachu v0.2.0 + galaxy0 (DEseq adaptative fold change: 0.5; Adjustment: 0.05; MAD: 0), using read coverages for the starting R_0_ library and for either the _Bs_R_14_ or _Ec_R_10_ replicates. Only overlapping (matching) peaks detected with both algorithms were considered for further analyses. Matching peaks and other types of overlaps (e.g. between peaks and genes, promoters, or terminators) were obtained with Bedtools Intersect intervals v2.31.0. The genomic coordinates of the *Rut* peaks identified with MACS2—which consistently yielded narrower peaks than the corresponding PEAKachu calls (Supplementary Fig. S1B)—were used for downstream sequence analyses. Note that the random priming and PCR amplification steps used to generate the starting gDNA fragment library may cause validated *Rut* peaks to extend beyond the actual *Rut* site boundaries (Supplementary Fig. S1C). A control pool of 5000 *Rut*-less sequences was built by sampling 130 nt-long regions of the *B. subtilis* 168 genome that were not intersecting with *Rut* peaks on either strand using the seqtk_sample v1.4 utility of Galaxy. Genomic regions unsuitable for H-SELEX analysis (not covered by the R_0_ sample) represented only 0.2% of the *B. subtilis* 168 genome.

### Duplex unwinding kinetics

Duplex substrates were prepared by hybridizing ^32^P-labeled transcripts (from a given round library) with the SEL oligonucleotide, as described [10]. The ^32^P-labeled duplexes were purified by immobilization on magnetic streptavidin Dynabead T1 beads, following the protocol described in the H-SELEX enrichment section above. Bead-tethered duplexes (5 nM) were mixed with Rho hexamers (20 nM) in helicase buffer and pre-incubated under shaking (300 rpm) for 5 min at 37°C. Then, 1 mM MgCl_2_, 1 mM ATP, and 400 nM oligo TRAP were added to the helicase mixture before incubation at 37°C under shaking (300 rpm). Reaction aliquots were taken at various times and mixed with one volume of 2× quench buffer (40 mM EDTA, 2% SDS, 300 mM sodium acetate, and 8% Ficoll-400) before loading on a 9% PAGE gel that contained 1× TBE buffer (89 mM Tris-borate, 2 mM EDTA, pH 8) and 0.5% sodium dodecyl sulfate (SDS). Detection and quantification of gel bands were performed by phosphorimaging with a Typhoon FLA-9500 instrument equipped with ImaqeQuant TL v8.1 software, as described [33, 34]. Burst amplitudes were determined by assuming, as first approximation, that Rho-dependent unwinding of the library duplexes follows pseudo-first order kinetics [10].

### Bacterial cultures and phenotypic analyses

Bacterial cultures were routinely grown in liquid or on solid Lysogeny broth (LB) medium. Standard protocols were followed for the transformation of *E. coli* and *B. subtilis* competent cells. Optical density (OD_600_) of bacterial cultures was measured with a Libra S11 Visible Spectrophotometer (Biochrom). When required, antibiotics were added at the following concentrations: erythromycin, 0.5 μg/ml; phleomycin, 3 μg/ml; spectinomycin, 100 μg/ml; and kanamycin, 5/ml for *B. subtilis*; or ampicillin, 100 μg/ml for *E. coli*.

To assess the formation of the red, insoluble pulcherrimin–iron complex, 2 μl of overnight cultures were spotted onto MSgg agar plates [35] and incubated at 30°C. Each assay included three independent isogenic cultures and at least three independent experiments were conducted. Images were acquired after 24, 48, and 72 h using a Samsung Galaxy Tab E–SM-T560 instrument.

For inhibition assays, *B. subtilis* and target strains *Bacillus thuringiensis* var. *israelensis* and *Bacillus weihenstephanensis* KBAB4 cells were grown to OD_600_ 1.1–1.2 in tryptic soy (TS) broth (BioMérieux, France) at 30°C. For lawn formation, ∼5 × 10^6^ of target cells were spread onto 1.5% agar TS plates. Then, 2 μl of *B. subtilis* cells were directly spotted onto the *B. thuringiensis* or *B. weihenstephanensis* lawn. At least three independent experiments were conducted, each including two independent isogenic *B. subtilis* cultures. Images were acquired after overnight incubation of the plates at 30°C.

Live-cell array experiments were performed in transparent, chemically defined MS medium [25] or SMS medium (14 g/l K_2_HPO_4_, 6 g/l KH_2_PO_4_, 2 g/l NH_4_SO_4_, and 1 g/l C_6_H_5_Na_3_O_7_·2H_2_O), supplemented with 6 mM of MgSO_4_, 0.5% glucose, 0.01% casein hydrolysate (Oxoid), 0.2% yeast extract (Diffco), and 0.005% L-tryptophan. *B. subtilis* cells bearing *pksD-gfp* or *dprA∼gfp* reporter fusions were first grown in LB medium to mid-exponential phase (OD_600_ 0.4–0.5), then centrifuged and resuspended in fresh MS or SMS medium, respectively, to an OD_600_ of 1.0. The pre-cultures were subsequently diluted to OD_600_ = 0.025 in the respective medium and distributed (150 μl per well) into flat-bottomed 96-well microtiter plates (Cellstar; Greiner Bio-One). Plates were incubated at 37°C with constant shaking in a Synergy 2 Multi-mode microplate reader (BioTek Instruments). Fluorescence (excitation filter: 485/20 nm; emission filter: 528/20 nm) and OD_600_ were measured at 5-min intervals. GFP expression levels were normalized for background fluorescence by subtracting values obtained from parental BsB1 (WT) or BRL1 (Δ*rho*) strains cultivated in independent wells of the same plate column. All experiments were repeated at least three times with consistent results. Data from representative experiments (mean ± standard deviation; *n* = 5) are presented in Figs 5B and 6E.

### Strain construction

Insertion of intrinsic transcription terminators (ITs) downstream of the *sucD* gene and deletion of the putative *Rut*_pks_ site in the noncoding *pksC-pksD* intergenic region of the BsB1 chromosome were performed by scarless gene replacement using the thermo-sensitive shuttle vector pMAD [36]. To insert ITs, primer pairs veb734/veb735, veb876/veb946, and veb867/veb947 were used to amplify, respectively, ITs from plasmid pMutin [37] and two flanking fragments from the *sucD–dprA* region of the BsB1 chromosome. The resulting PCR fragments contained overlapping sequences and were joined by overlap extension PCR using primers veb946 and veb867. The assembled fragment was cloned between the EcoRI and BamHI sites of the pMAD plasmid, yielding plasmid pBRL1408. This plasmid was transformed into BsB1 cells and used for chromosomal integration and subsequent markerless excision following the standard pMAD protocol [36]. The successful insertion of the ITs and the integrity of the modified *sucD*–*dprA* locus in the resulting strain BRL1412 were confirmed by PCR and DNA sequencing.

To delete the *Rut*_pks_ region, partially overlapping oligonucleotides veb977 and veb978, which match the extremities of the chromosomal fragment containing *Rut* sites Ec_R10_P_427 and Bs_R14_P_81 (Supplementary Table S4), were used in PCR in pairs with veb976 and veb979, respectively, using the BsB1 chromosome as the template. The resulting PCR fragments were joined by overlap extension PCR using primers veb976 and veb979, and the assembled product was cloned between the SalI and BamHI sites of the pMAD vector. The resulting plasmid pBRL1450 was transformed in BsB1 cells and subjected to the standard pMAD-based scarless allelic replacement procedure. The composition of the modified *pksC-pkSD* intergenic region in the resulting BRL1454 strain was confirmed by PCR and DNA sequencing.

To test the activity of the intragenic RDTT site in *dprA*, the *gfp* gene from plasmid pCVO119m (encoding monomeric GFP) was fused to the 3′-end of *dprA* such that the *gfp* start codon (ATG) overlapped with the *dprA* stop codon (TGA), forming an ATGA sequence. This arrangement allowed for translational read-through and expression of GFP from the extended *dprA-gfp* transcript. To construct the fusion, the *gfp* gene was PCR amplified from pCVO119m using primers veb740 and veb741 while the 3′-terminal segment of *dprA* gene was amplified using primers veb872 and veb874. The two PCR products were fused by overlap extension PCR using primers veb872 and veb741. The resulting fragment was cloned between the SalI and EcoRI sites of plasmid pBRL892, a derivative of pCVO119 from which the *gfp* gene had been removed by digestion with SphI and XhoI, blunting with T4 DNA polymerase, and self-ligation. The resulting plasmid pBRL1292 was integrated by single crossover into the chromosomes of BsB1 and BRL1412 competent cells, thereby generating a *dprA∼gfp* translational fusion in the WT *sucCD-dprA* locus and in the modified *sucCD-IT-dprA* locus, respectively.

To analyze RDTT in the *pksC-pksD* region, the *gfp* gene was inserted at the *pksD* locus of BsB1 and BRL1454 strains by replacing *pksD* at its start codon. DNA fragments upstream of the *pksD* gene were PCR amplified from the BsB1 and BRL1454 chromosomes using primers veb982 and veb983, and fused to the *gfp* gene (amplified from plasmid pCVO119m, as described above) by overlap extension PCR using primers veb983 and veb741. The resulting PCR products were cloned between the EcoRI and SalI sites of plasmid pBRL892, yielding plasmids pBRL1484 and pBRL1485. These plasmids were integrated by single crossover recombination into the chromosomes of BsB1 and BRL1454 competent cells, respectively. All *gfp* reporter strains constructed by single crossover integration were verified by PCR to contain a single chromosomal copy of the integrated plasmids, as described previously [25].

To restore bacillaene production in *B. subtilis* BsB1 and its derivative strains, an active allele of the *sfp* gene was PCR amplified from the chromosome of the nondomesticated *B. subtilis* NCBI 3610 strain using primers veb989 and veb990. The PCR product was cloned between the BamHI and SalI sites of plasmid pBRL892, yielding plasmid pBRL1452. This plasmid was transformed into BsB1, BRL1, and BRL1454 competent cells. Spectinomycin-resistant transformants were screened for the presence of the *sfp^+^* allele by PCR using primers veb991 and veb992, which anneal to the chromosomal region upstream of *sfp* and to the vector backbone of pBRL1452, respectively. Correct integration was further confirmed by DNA sequencing.

### Northern blot analyses

RNA was isolated from mid-log phase *B. subtilis* cells—grown in rich controlled medium (MD medium containing 10.7 mg/ml K_2_HPO_4_, 6 mg/ml KH_2_PO_4_, 1 mg/ml sodium citrate, 0.5% w/v malate, 11 μg/ml ferric ammonium citrate, 2 mg/ml aspartic acid neutralized to pH 7 with KOH, and 0.36 mg/ml MgSO_4_) supplemented with 0.5% malate as carbon source—by the RNAsnap method, as described previously [38]. Typically, 5 μg of RNA was run on 1% agarose in 1× TBE buffer and transferred to Hybond-N + membranes (Cytiva) by capillarity. Hybridization was performed using either 5′-labeled oligonucleotides labeled with [γ-^32^P]-ATP and T4 polynucleotide kinase, following manufacturer’s instructions or a riboprobe transcribed from a PCR-amplified DNA template containing a T7 promoter and labeled during T7 transcription with [α-^32^P]-UTP. Hybridization was carried out at 42°C (for oligonucleotide probes) or 68°C (for riboprobes) for a minimum of 4 h in UltraHyb hybridization buffer (Ambion). Membranes were washed twice in 2× SSC/0.1% SDS (once rapidly at room temperature and once for 10 min at 42°C) and then three times for 10 min in 0.2× SSC/0.1% SDS at room temperature.

### Statistical analyses

Mann–Whitney *U* tests and permutation tests were performed using dedicated Python functions from the Scipy and Numpy libraries. Chi-square and Fisher’s exact tests were performed online (www.socscistatistics.com). ANOVA tests were performed with Kaleidagraph v4.1.1 (Synergy software). Reported *P*-values are for two-sided tests. The number of experimental replicates (*n*) is indicated in methods or figure legends.

## Results and discussion

### Genome-wide mapping of putative *B. subtilis*rut sites using H-SELEX

In H-SELEX, we leverage the activity of the Rho helicase to iteratively enrich RNA libraries in specimens containing *Rut* sequences (Fig. 2A) [10].

To this end, we first purified enzymatically active *B. subtilis* Rho (_Bs_Rho) to near homogeneity (Supplementary Fig. S2A), using a native purification protocol (see methods) that is less disruptive—based on steady-state ATPase rates (Supplementary Fig. S2B)—than the previously published denaturation-renaturation protocol [16]. Using a model RNA–DNA duplex, we determined that _Bs_Rho possesses helicase activity, though it is weaker than the activity of *E. coli* Rho (_Ec_Rho) (Supplementary Fig. S2C). Moreover, _Bs_Rho-mediated duplex unwinding was enhanced at basic pH whereas _Ec_Rho activity remained largely unaffected (Supplementary Fig. S2C). Based on these findings, we performed the H-SELEX selection steps at pH 9.0.

We used a four-channel liquid handling robot to carry out the H-SELEX enrichment cycles (see “Materials and methods” section), enabling the simultaneous processing of four samples. This setup allowed us to compare the screening selectivities of _Bs_Rho and _Ec_Rho while also conducting each screening in duplicate. To generate the initial H-SELEX library, we amplified gDNA from *B. subtilis* 168 *trp*^+^ strain BsB1 using semi-randomized primers, thereby producing gDNA fragments [∼30 to ∼300 base pairs (bp)] that covered >98% of the genome. The gDNA fragments are flanked by constant sequences compatible with the transcription, immobilization, and Reverse Transcriptase PCR (RT-PCR) amplification steps of H-SELEX (Fig. 2A) [10]. We transcribed this initial gDNA fragment library (R_0_ library) and converted it into a library of biotinylated RNA–DNA duplexes immobilized on streptavidin beads. This duplex library was then exposed to either the _Bs_Rho or _Ec_Rho helicase. RNA species released in the supernatant upon Rho-mediated duplex unwinding were collected for RT-PCR amplification (Fig. 2A), generating the next-round gDNA fragment libraries enriched in *Rut* sequences (_Bs_R_1a_ and _Bs_R_1b_ for _Bs_Rho; _Ec_R_1a_ and _Ec_R_1b_ for _Ec_Rho). This enrichement process was repeated iteratively under increasing stringency, achieved by progressively reducing the helicase reaction time from 10 min in round 1 to 20 s in the final H-SELEX rounds (Supplementary Table S3). We observed a gradual increase in helicase-induced responses across successive rounds for both _Bs_Rho- and _Ec_Rho-enriched libraries (Fig. 2B). Maximal enrichment—estimated from helicase reaction burst amplitudes [10]—was reached more rapidly with _Ec_Rho (round 10) than with _Bs_Rho (round 14) (Fig. 2C). Even with the maximally enriched libraries, duplex unwinding remained more efficient and faster with _Ec_Rho than with _Bs_Rho (Fig. 2B–D), mirroring observations with our model RNA-DNA substrate (Supplementary Fig. S2C). Thus, even with optimized *Rut*-bearing substrates, _Bs_Rho appears to be an intrinsically less efficient helicase than _Ec_Rho.

**Figure 3.**
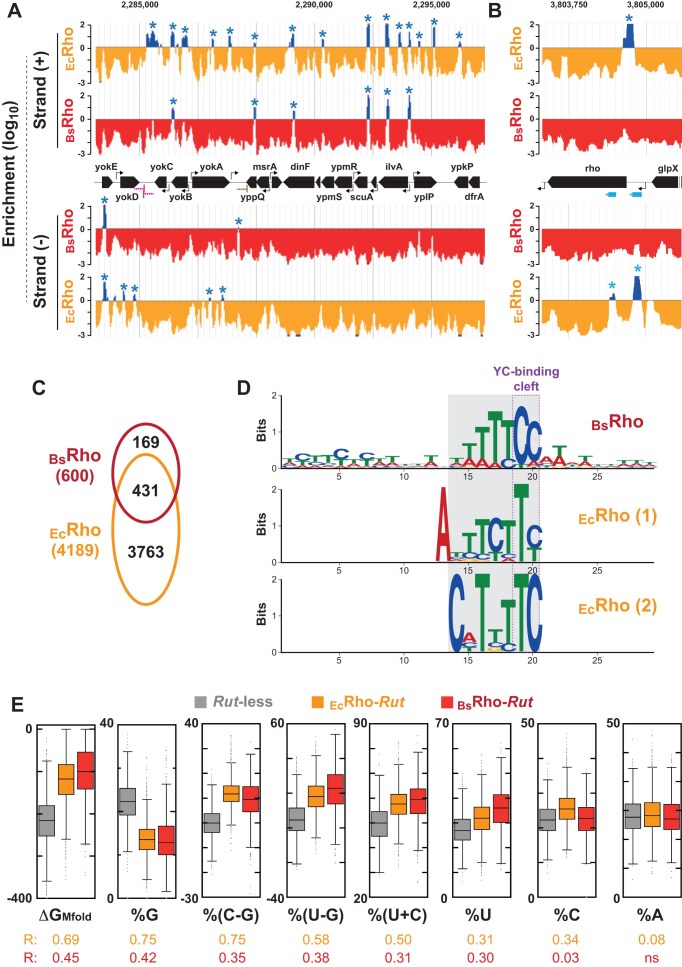
Detection of discrete *Rut* peaks along the *B. subtilis* genome. (**A**and **B**) Examples of R_10_/R_0_ (for _Ec_Rho) and R_14_/R_0_ (for _Bs_Rho) enrichment profiles (log_10_ scale) for selected regions of the *B. subtilis* genome (different scales along *x*-axis). Positive values are in dark blue while negative values are in orange (_Ec_Rho) or red (_Bs_Rho). Blue asterisks mark *Rut* peaks validated by peak calling analysis of replicate samples (see “Materials and methods” section). Cyan rectangles highlight sense *Rut* sites likely involved in Rho-dependent regulation of the *rho* gene [16]. (**C**) Venn diagram of validated *Rut* peaks obtained with _Ec_Rho and _Bs_Rho. (**D**) Consensus motifs identified for _Bs_Rho-*Rut* (385 sites; *E*-value = 4.5 × 10^−47^) and _Ec_Rho-*Rut* (2250 sites; *E*-val = 1.9 × 10^−69^ for motif 1 and 1165 sites; *E*-val = 2.6 × 10^−17^ for motif 2) using differential MEME analysis [41]. (**E**) Box plots for selected sequence descriptors for _Bs_Rho-*Rut*, _Ec_Rho-*Rut*, and *Rut*-less sequence pools. Δ*G*_Mfold_ represents the free energy of secondary structure formation computed with Mfold and normalized per kilobase. Effect sizes (R) for Mann–Whitney *U* tests comparing _Bs_Rho-*Rut* versus *Rut*-less (orange) or _Bs_Rho-*Rut* vs. *Rut*-less (red) sequences are displayed below the box plots.

To identify putative *Rut* sites across the *B. subtilis* genome and evaluate the selectivity of the _Bs_Rho enzyme, we deep-sequenced the initial gDNA fragment library (R_0_) and the final libraries enriched with _Bs_Rho (_Bs_R_14a,b_) and _Ec_Rho (_Ec_R_10a,b_). Comparison of the final and starting libraries revealed hundreds of discrete H-SELEX enrichment peaks (Fig. 3A,B and Supplementary Fig. S3, in blue), hereafter referred to as *Rut* peaks to follow the nomenclature of previous work [10]. Our conservative detection pipeline selected only replicate-independent *Rut* peaks detected with two distinct peak calling algorithms, MACS2 [39] and PEAKachu [40], for further analysis. This analysis identified 4 189 *Rut* peaks (∼497/Mb) from _Ec_Rho enrichment and 600 *Rut* peaks (∼71/Mb) from _Bs_Rho enrichment (Fig. 3C; see Supplementary Table S4 for lists of *Rut* peaks). For comparison, H-SELEX screening of the *E. coli* MG1655 genome with _Ec_Rho yielded a *Rut* peak density of ∼281/Mb [10]. These results suggest that the *B. subtilis* transcriptome may contain a higher density of suitable *Rut* sites than *E. coli*, but that the _Bs_Rho enzyme has evolved to fine-tune target selection through enhanced specificity. Alternatively, _Bs_Rho’s weaker helicase activity (Fig. 2B–D) may have led to H-SELEX over-enriching the most efficient *Rut* sequences at the expense of those requiring specific conditions or cofactors absent from our assay.

This hypothesis is supported by the H-SELEX profiles of the *B. subtilis rho* gene, which is known to be regulated by Rho-dependent transcription attenuation [16]. While no *Rut* peaks were detected in the *rho* region upon enrichment with _Bs_Rho, two candidate *Rut* sites were found with _Ec_Rho (Fig. 3B, cyan arrows), at expected locations in *rho*’s 5′UTR and upstream section of its coding sequence (CDS) [16]. Based on these observations, we propose that (i) _Bs_Rho-*Rut* peaks identified with _Bs_Rho define the core set of *B. subtilis Rut* sites, and (ii) at least some _Ec_Rho-*Rut* peaks detected exclusively with the more permissive _Ec_Rho enzyme represent genuine *B. subtilis Rut* sites, albeit ones requiring particular conditions or _Bs_Rho cofactor(s).

### *B. subtilis* Rut sequences lack stable RNA structures and feature short U/C-rich anchor motifs

The majority (72%) of _Bs_Rho-*Rut* peaks co-localize with an _Ec_Rho-*Rut* peak along the *B. subtilis* genome strands (Fig. 3A and C, and Supplementary Fig. S3), further supporting that both enzymes share similar substrate requirements despite their different helicase efficiencies. The main populations of _Bs_Rho-*Rut* and _Ec_Rho-*Rut* peaks have an average length of ∼100 nt (Supplementary Fig. S4A, left graph), likely reflecting the optimal *Rut* length for forming a catalytically active Rho-RNA complex (Fig. 1). However, shorter *Rut* sequences (∼50 nt) can also form productive complexes (and may be functional *in vivo*; see Supplementary Fig. S5), and this occurs more frequently with _Bs_Rho than with _Ec_Rho, in particular for “orphan” *Rut* peaks (Supplementary Fig. S4A, right graph). The latter also have slightly distinct C residue contents (Supplementary Fig. S4B). Moreover, _Ec_Rho appears to accommodate a broader range of *Rut* site sizes (Supplementary Fig. S4A), possibly due to a greater ability to unfold or bind secondary RNA structures. In support of this proposal, _Ec_Rho-*Rut* peak sequences generally encode more stable RNA structures than _Bs_Rho-*Rut* peak sequences (Δ*G*_Mfold_ = [−14.8 ± 10] kcal/mol and [−9.7 ± 8] kcal/mol, respectively; Mann–Whitney *U* test, *P*-value < 10^−4^), based on Mfold computations [42].

To analyze the sequence features of the _Bs_Rho-*Rut* and _Ec_Rho-*Rut* peaks further, we generated a pool of *Rut*-less control sequences (130 nt; 5000 specimens) randomly selected in genomic regions devoid of _Bs_Rho-*Rut* and _Ec_Rho-*Rut* peaks. We then conducted differential MEME analyses [41], comparing _Bs_Rho-*Rut* and _Ec_Rho-*Rut* peak sequences against this control pool. These analyses identified similar pyrimidine-rich consensus motifs in _Bs_Rho-*Rut* (one motif) and _Ec_Rho-*Rut* (two motifs) peak sequences (Fig. 3D, gray box), likely reflecting comparable requirements for efficient RNA–PBS interactions on each Rho subunit (Fig. 1B). In the case of _Bs_Rho-*Rut* peaks, the sequence consensus weakly extends on both sides of the pyrimidine-rich motif (Fig. 3D, top), suggesting that the sequence and spatial constraints for RNA-PBS interactions across adjacent Rho subunits are stricter for _Bs_Rho than for _Ec_Rho.

Next, we computed sequence descriptors, such as nucleotide percentages or free energy for secondary structure formation (Δ*G*_Mfold_), for each _Bs_Rho-*Rut*, _Ec_Rho-*Rut*, and control sequence. We then ranked significant descriptors (Mann–Whitney *U* test, *P*-value ≤ 0.05) based on their standardized effect size *R*, with the most discriminative descriptors having the highest R (Supplementary Table S5). Comparisons between the _Bs_Rho-*Rut* and _Ec_Rho-*Rut* datasets revealed small effect sizes (*R* < 0.21), further suggesting that _Bs_Rho and _Ec_Rho have similar substrate requirements. In contrast, effect sizes were larger (up to *R* ∼0.75) when comparing *Rut* peak datasets to the *Rut*-less control pool (Supplementary Table S5). The most discriminative descriptors support that *Rut* sequences are generally richer in pyrimidine residues, poorer in guanosine residues, and less prone to form stable RNA secondary structures than *Rut*-less sequences (Fig. 3E and Supplementary Table S5). Moreover, significant U > G and C > G biases in the _Bs_Rho-*Rut* and _Ec_Rho-*Rut* sequences (Fig. 3E) suggest that the empirical rule of Rho-dependent terminators preferentially occurring in C > G “bubbles” [13, 14, 43] extends to *B. subtilis*. However, no combination of descriptors was sufficient to reliably distinguish *Rut* from *Rut*-less sequences (Supplementary Fig. S6), indicating that key *Rut* features remain elusive and continue to limit the accuracy of computational RDTT predictions [14, 44].

### Skewed genomic distribution of Rut sites underscores Rho’s role in curbing antisense transcription

**Figure 4.**
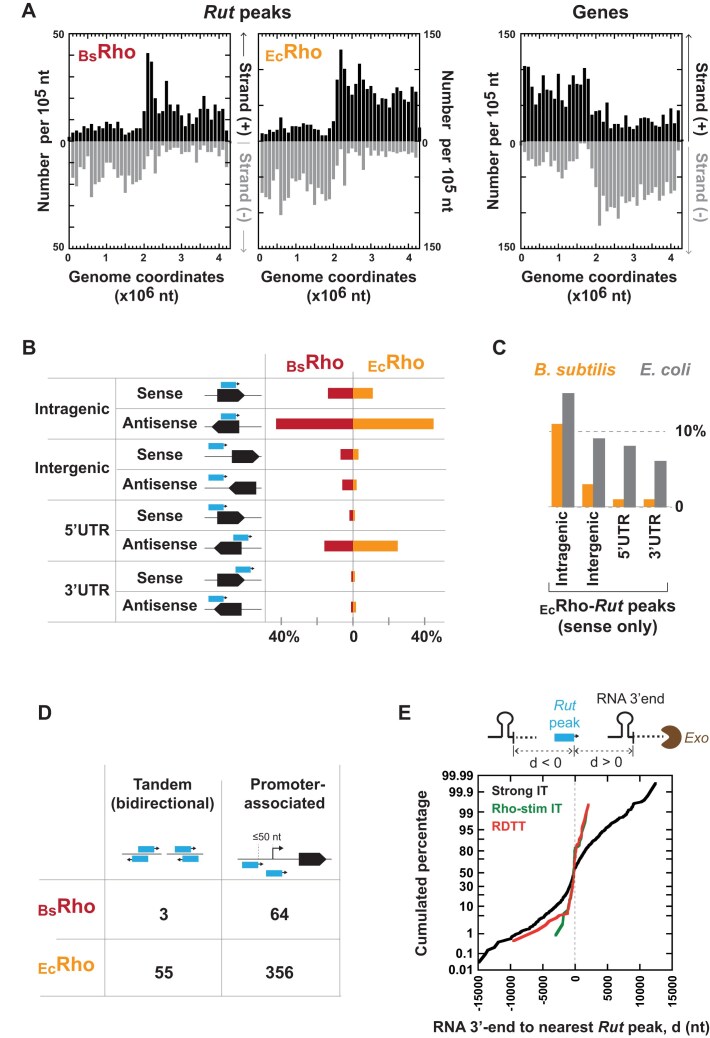
Main features of *Rut* peaks. (**A**) The distribution of *Rut* peaks along the *B. subtilis* genome inversely mirrors the distribution of genes. (**B**) Categories of *Rut* peaks (blue arrows) and their respective proportions. Red and orange bars indicate the percentages of *Rut* peaks obtained with _Bs_Rho and _Ec_Rho, respectively. (**C**) *Rut* peaks in sense orientation are less frequent in *B. subtilis* (orange bars) than in *E. coli* (gray bars). (**D**) Numbers of *Rut* peaks arranged in bidirectional tandem pairs or near promoters. (**E**) Probability plot of the shortest distances between *Rut* peaks and transcript 3′ ends attributed to strong IT (black curve), Rho-stimulated IT (green curve), or RDTT (red curve). The reference point (0) is the downstream edge of the nearest *Rut* peak, as depicted in the diagram above the graph. Negative distances (*d* < 0) may indicate 3′-to-5′ exonucleolytic trimming beyond the *Rut* site.

Next, we examined the distribution of _Bs_Rho-*Rut* and _Ec_Rho-*Rut* peaks along the *B. subtilis* genome. Similar to what was observed for *E. coli* [10], *Rut* peaks tend to be more abundant in prophages (Supplementary Fig. S7A), substantiating the notion that Rho contributes to control transcription of xenogeneic DNA [45–47]. Moreover, the genomic distribution of the *Rut* peaks is skewed in an almost perfectly inverse manner when compared to the distribution of the *B. subtilis* genes (Fig. 4A; compare with the situation in *E. coli* in Supplementary Fig. S7B). Consistent with this observation, most *Rut* peaks (∼80%) are positioned antisense to genes (Fig. 4B and Supplementary Table S4). This predominant arrangement is independent of the Rho enzyme used for H-SELEX enrichment (Fig. 4A and B; Fisher’s exact test, *P*-value = 0.16) and is observed for both xenogeneic and core genome regions, aligning with the increase in antisense transcription seen in these regions in *B. subtilis* Δ*rho* cells [21, 23] (see representative examples in Supplementary Fig. S8).

While these findings support Rho’s function in silencing pervasive antisense transcription, conditional antisense read-through at RDTT terminators may contribute to *B. subtilis* adaptive programs. Notably, antisense read-through in Δ*rho* cells correlates with the unscheduled expression of sporulation genes, as observed in previous transcriptome profiling studies [22, 23]. We identified antisense *Rut* peaks (as defined in Fig. 4B), often clustered upstream from sporulation-related loci, including *sspC, sspM, yraED, ysnD*, and the *gerPA-PB-PC-PD-PE-PF* operon (Supplementary Table S4 and Supplementary Fig. S8). This supports that Rho helps prevent premature sporulation under noninducing conditions [21, 23] and underscores the dual role of RDTT in both constitutive and adaptive gene regulation.

### Regulation of *B. subtilis* genes and operons by internal RDTT signals

The proportion of putative *Rut* sites potentially involved in a more direct “sense” regulation of genes is significantly lower in *B. subtilis* than in *E. coli* (Fig. 4C; Fisher’s exact test, *P*-value < 10^−3^). Notably, we did not find *Rut* peaks associated with metabolite-sensing riboswitches or cold shock genes (Supplementary Fig. S9A and B), in contrast to observations in other bacteria [10, 29, 48]. This is consistent with the fact that metabolite-sensing riboswitches in *B. subtilis* rely primarily on the restructuring of IT motifs, a mechanism less common in phylogenetically divergent species such as *E. coli* [49, 50]. Nonetheless, we detected *Rut* peaks in three *B. subtilis* operons/genes regulated by protein-dependent attenuation mechanisms (Supplementary Fig. S9C) [49], including the *trpEDCFBA* operon where regulation by the TRAP protein and excess tryptophan involves translational repression and Rho-dependent transcriptional polarity [51].

We also did not find *Rut* peaks in genes encoding functionally annotated noncoding RNAs (ncRNAs) (e.g. SSrA, scr, RoxS, 6S RNAs, RNA component of RNase P, ncRNAs of toxin–antitoxin systems) or their respective targets, as defined in the *Subti*Wiki database [52]. However, we detected *Rut* peaks in nearly half (76/153) of the genome regions previously categorized as encoding “independent,” presumably noncoding, transcripts (Supplementary Table S6). These ncRNAs are expressed primarily under stress or sporulation conditions, under the control of their own promoters and alternative sigma factors [21, 53]. Notably, a substantial subset (34/76) is upregulated in Δ*rho* cells during exponential growth in rich medium (Supplementary Table S6) [23]. These findings suggest that Rho contributes to the biogenesis of ncRNAs involved in the *B. subtilis* adaptation and differentiation programs.

Among the *Rut* peaks indicative of direct Rho-dependent regulation of protein-coding genes, we identified an _Ec_Rho-*Rut* peak within the CDS of *kinB* (Supplementary Table S4), a gene strongly upregulated in Δ*rho* cells (∼7 fold) during exponential growth in LB [23]. This _Ec_Rho-*Rut* peak is positioned just upstream from a marked downshift in the *kinB* transcription profile of WT cells (Supplementary Fig. S10) [21], within a region of the *kinB* CDS previously shown to be necessary for RDTT [22]. These observations support the idea that RDTT can initiate from intragenic positions in *B. subtilis* [23]. Interestingly, some intragenic *Rut* peaks are present in long polycistronic transcription units, suggesting that expression of the downstream operon genes could depend on cellular Rho levels or other factors regulating RDTT. For instance, an _Ec_Rho-*Rut* peak is located in the upstream section of *albF* (Supplementary Table S4), which is part of the *albABCDEFG* operon involved in subtilosin production [54]. Consistently, in the Δ*rho* mutant, the distal *albE/F/G* genes are upregulated (up to ∼7-fold) during exponential growth in LB, whereas the proximal *albA/B/C/D* genes show minimal changes (<1.5-fold change) [23]. Conversely, in a strain overexpressing Rho, *albE/F/G* are more strongly repressed (up to ∼8-fold) than *albA/B/C/D* (up to ∼1.5-fold) when compared to the isogenic WT strain [23]. Furthermore, subtilosin and other antimicrobial compounds (see below) are produced when WT *B. subtilis* enters stationary phase, coinciding with decreased *rho* mRNA and Rho protein levels [21, 23, 25]. Another example of intragenic *Rut* site regulating the production of a secondary metabolite is located in the first gene of the *ntdABC* operon (Supplementary Table S4), responsible for the biosynthesis of kanosamine [55]. While this operon is normally dormant in *B. subtilis*, it can be activated by a single-point RNAP mutation [55] as well as by *rho* inactivation [22, 23], further implicating intragenic RDTT in adaptive gene regulation.

**Figure 5.**
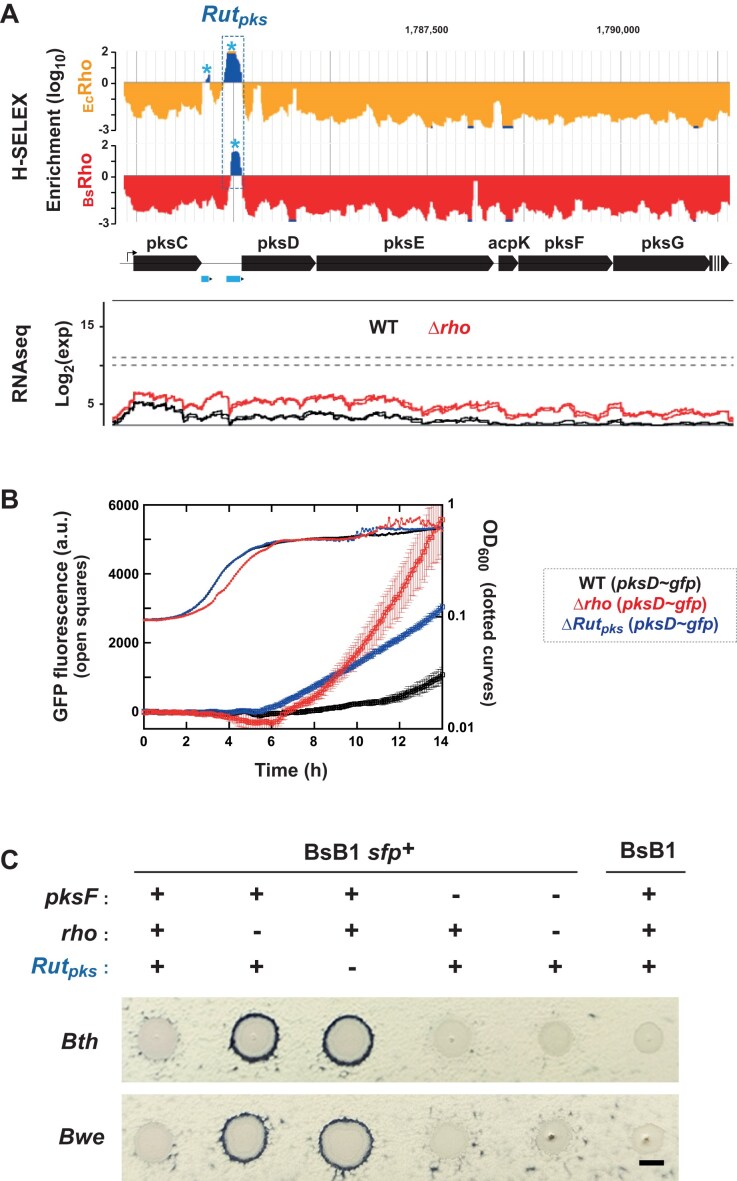
Rho-mediated regulation of the expression of the bacillaene-encoding gene cluster. (**A**) H-SELEX enrichment (top) and transcription (RNAseq) profiles for the WT and Δ*rho* strain (bottom) in the upstream region of the operon. Blue asterisks mark validated *Rut* peaks. RNAseq profiles [23] are snapshots from the Genoscapist website (https://genoscapist.migale.inrae.fr/) [59]. A putative *Rut* site mediating *pks* regulation by Rho is detected in both _Bs_Rho and _Ec_Rho H-SELEX profiles (*Rut_pks_*). For simplicity, antisense profiles are not shown. (**B**) Time-courses of normalized GFP fluorescence and optical density at 600 nm (OD_600_) observed for the strains listed next the graph. (**C**) Spot plating assay of the inhibitory activity of *B. subtilis* BsB1 *sfp*^+^ against *B. thuringiensis var. israelensis* (*Bth*) and *B. weihenstephanensis* KBAB4 (*Bwe*). Zones of clearing were compared between BsB1 *sfp*^+^ and its derivatives BsB1 Δ*rho sfp*^+^, BsB1 Δ*Rut_pks_ sfp*^+^, BsB1 Δ*pksF sfp*^+^, and BsB1 Δ*rho* ΔpksF *sfp*^+^. The WT *B. subtilis* BsB1 strain was used as negative control. Images were taken after overnight incubation at 30°C. The experiments were reproduced at least three times and representative results are shown. The black scale bar corresponds to 5 mm.

We also identified sense-oriented *Rut* peaks in intergenic regions of polycistronic operons (Supplementary Table S4), which may serve similar regulatory functions. For example, _Bs_Rho-*Rut* and _Ec_Rho-*Rut* peaks were found between *pksC* and *pksD* in the long *pksCDE-acpK-pksFGHIJLMNR* operon (Fig. 5A) directing the biosynthesis of bacillaene, a polyene antibiotic with broad activity against bacteria and fungi [56, 57]. Consistently, in Δ*rho* cells during exponential growth in LB, gene upregulation was more pronounced downstream of the *Rut* peaks (∼5-fold for *pksD* and ∼16-fold for *pksE*) than upstream (∼2-fold for *pksC*) (Fig. 5A) [23].

To test whether the putative *Rut* sites identified by H-SELEX are involved in Rho-dependent regulation of the *pks* operon, we deleted the region corresponding to the overlapping _Bs_Rho-*Rut* and _Ec_Rho-*Rut* peaks located in the *pksC-pksD* intergenic region (Fig. 5A, dotted box) from the *B. subtilis* BsB1 chromosome. We first assessed the effect of this deletion (hereafter referred to as Δ*Rut_pks_*) using *gfp* reporter strains in which the *gfp* gene was inserted at the start codon of *pksD* (see “Materials and methods” section). Live-cell array experiments revealed that the activity of this *pksD*-*gfp* transcriptional fusion is significantly higher in cells harboring the Δ*Rut_pks_* or Δ*rho* deletion than in the parental (WT) BsB1 strain (Fig. 5B), supporting that RDTT originating from the *Rut_pks_* site contributes to regulate the *pks* operon. The higher GFP fluorescence observed in Δ*rho* cells compared to Δ*Rut_pks_* cells may be explained by the presence of an additional upstream *Rut* site (i.e. the smaller _Ec_Rho-*Rut* peak in Fig. 5A). Alternatively, this difference could reflect indirect effects of Rho inactivation on the regulation of the *pksC* promoter by global regulators CodY and AbrB [58].

We further investigated whether Rho-mediated regulation of the *pks* operon affects bacillaene production *in vivo*. Because *B. subtilis* BsB1 carries a nonfunctional *sfp* allele [60], which encodes an inactive variant of the phosphopantetheinyl transferase required for bacillaene synthesis [61], we restored *sfp* function by introducing the WT allele at its native locus in BsB1 and derivative strains. Using a spot inhibition assay [57, 62], we found that both Δ*rho sfp*^+^ and Δ*Rut_pks_ sfp*^+^ strains produced more pronounced inhibition halos than the parental BsB1 *sfp*^+^ strain on lawns of bacillaene-sensitive *B. thuringiensis* var*. israilensis* (*Bth*) and *B. weihenstephanensis* KBAB4 (*Bwe*) (Fig. 5C). The absence of marked halos for isogenic strains deleted for *pksF*, which encodes a key ketosynthase component of the Pks enzymatic complex [57] confirmed that the observed bacteriostatic activities depend on bacillaene (Fig. 5C). These results unambiguously demonstrate that bacillaene production is, at least partly, regulated by Rho action at the intergenic *pksC-pksD Rut_pks_* site.

Taken together, these findings support that RDTT from *Rut* sites located in both intra- and inter-genic regions of *B. subtilis* operons contribute to transcriptional polarity along the co-transcribed genes. While this resembles “textbook” Rho-dependent polarity in *E. coli*, it remains to be determined how this process is influenced by translation in *B. subtilis*, given the weaker coupling between transcription and translation in this species [63]. Previous attempts to address this relationship relied on artificial intragenic RDTT constructs [63], which may not fully recapitulate the features and requirements of natural intragenic terminators.

Other arrangements of *Rut* peaks are more intriguing and may have regulatory functions not yet fully characterized or comprehended. For example, ∼10% of *Rut* peaks are positioned just upstream from, or overlapping with, promoters (Fig. 4D and Supplementary Table S4). A similar proportion of promoter–proximal *Rut* peaks has been observed in *E. coli* [10], suggesting that this conserved feature may help control pervasive transcription or prevent transcriptional read-through into downstream transcription units. In *E. coli*, a promoter-proximal *Rut* site modulates expression of the downstream *cspI* transcription unit in a temperature-dependent manner [29]. In *B. subtilis*, promoter–proximal Rut peaks are often associated with genes regulated by both the vegetative sigma factor SigA and alternative sigma factors, typically involved in sporulation or stress responses (Supplementary Table S4). This suggests that RDTT from promoter-proximal *Rut* sites could contribute to adaptive gene expression in a Sigma-dependent manner, an intriguing hypothesis that remains to be tested.

Equally intriguing is a small subset of *Rut* peaks forming tandem pairs on opposite DNA strands (Fig. 4D and Supplementary Table S4). Bidirectional tandem *Rut* peaks were also observed in *E. coli* [10]. In *B. subtilis*, these tandem *Rut* peaks do not follow a strict localization pattern, occurring in both intra- and inter-genic regions, with nearby genes in either convergent or divergent orientation. Furthermore, ∼80% of the tandem *Rut* peaks are located in regions where there was no significant transcriptional increase for either DNA strand in the Δ*rho* strain under the tested conditions [21–23]. Thus, the functional significance of this specific arrangement of *Rut* sites remains to be determined.

### Evidence for 3′-to-5′ exonucleolytic trimming of the *B. subtilis* rho-dependent transcripts

To assess the relationship between *Rut* peaks and transcript 3′-ends attributed to *in vivo* transcription termination events [26], we measured the distance between each transcript 3′-end and the nearest *Rut* peak. This distance varies significantly depending on the type of termination event (χ^2^ test, *P*-value < 10^−4^). For strong IT events, 3′-ends are often very far from *Rut* peaks (Figs. 4E and Supplementary S11), consistent with the two being mechanistically unrelated. In contrast, for Rho-stimulated (otherwise weak) IT or RDTT events, transcript 3′-ends are in general much closer to *Rut* peaks (Fig. 4E and Supplementary S11), supporting a mechanistic link between them. Additionally, RDTT transcript 3′-ends can be located farther upstream from the nearest *Rut* peak (*d* < 0 in Fig. 4E and Supplementary S11) than Rho-stimulated IT transcript 3′-ends. This suggests that the terminator hairpins of weak IT signals provide some protection against 3′-to-5′ exonuclease trimming, whereas RDTT transcript 3′-ends are more extensively degraded until a stable RNA structure blocks exonuclease progression, as previously observed in *E. coli* [27]. This is consistent with the activity of PNPase, the primary 3′-to-5′ exonuclease in *B. subtilis*, which is impeded by RNA secondary structures [64].

**Figure 6.**
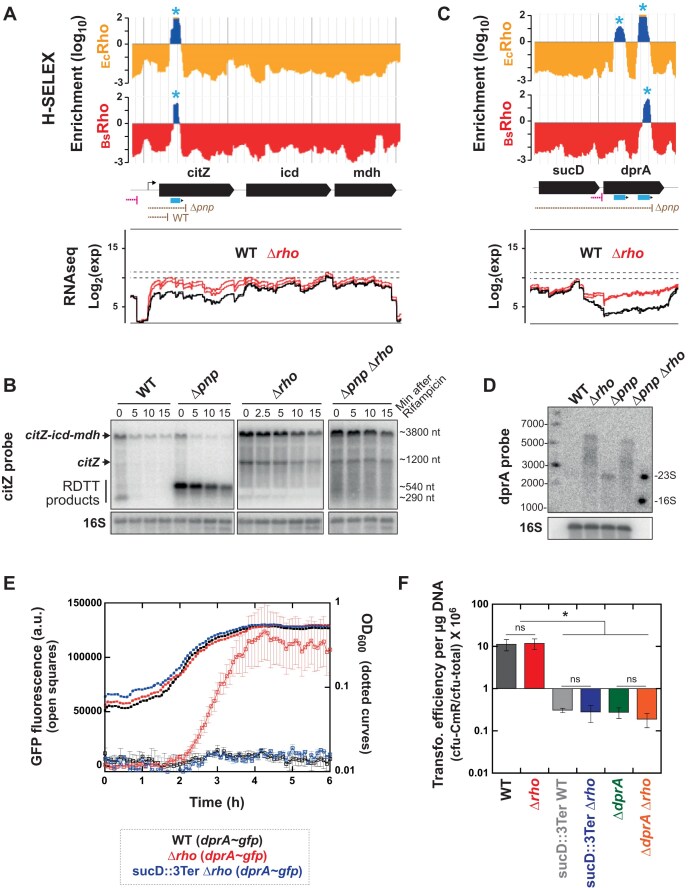
Representative examples of post-transcriptional processing of Rho-dependent transcripts. (**A**and **C**) H-SELEX enrichment (top) and RNAseq (bottom) profiles for the genomic regions of interest. Blue asterisks mark validated *Rut* peaks. Pink symbols show positions of transcript 3′-ends attributed to Rho-stimulated IT [26]. Brown symbols show positions of the 3′-ends detected by northern blots in the WT and Δ*pnp* strains. (**B**and **D**) Northern blot probing of the transcripts formed in WT *B. subtilis* and deletion mutant derivatives Δ*pnp*, Δ*rho*, and Δ*pnp* Δ*rho*. Panel (B) shows time courses of the stability of the various *citZ-icd-mdh* transcripts after inhibition of transcription with rifampicin. (**E**) Time-courses of normalized GFP fluorescence and optical density at 600 nm (OD_600_) observed for the strains listed below the graph. 3Ter refers to the insertion of the three strong IT terminators downstream of *sucD* (see the main text). (**F**) DNA transformation efficiencies for WT *B. subtilis* and mutant strains. Statistical significance was determined by ANOVA testing (*n* = 3; Bonferroni adjusted *P*-value: ns if *P* ≥ 0.05, * if *P* < 0.05).

Northern blot analysis of individual RDTT transcripts confirmed their post-transcriptional processing by PNPase. For example, we examined the expression of the *citZ-icd-mdh* transcription unit for which both H-SELEX (this work) and transcriptome profiling [21] hinted at regulation by Rho (Fig. 6A). Northern blots with an oligonucleotide probe targeting the upstream part of *citZ* detected two major transcript species in WT cells: a long species (∼3.8 kB) corresponding to the polycistronic *citZ-icd-mdh* mRNA and a much shorter one (∼290 ± 160 nt), likely representing a mature, processed form of *citZ* mRNA (Fig. 6B). This short, processed mRNA is degraded more rapidly than the *citZ-icd-mdh* mRNA following transcription inhibition by rifampicin (Fig. 6B). In a PNPase deletion mutant (Δ*pnp*), the short mRNA is no longer detected while a longer (∼540 ± 90 nt) transcript species becomes predominant (Fig. 6B). This new RNA species is highly stable in the Δ*pnp* background but is replaced by longer transcripts in a Δ*rho* Δ*pnp* strain (Fig. 6B), indicating that it is a direct product of RDTT. The length of this RNA product is consistent with RDTT occurring downstream of the *Rut* site identified by H-SELEX (+353 to +504 from the transcript start site; Fig. 6A).

We also examined the expression of the *dprA* gene, which encodes the DNA recombination mediator protein DprA, essential for efficient transformation of naturally competent *B. subtilis* cells by foreign DNA [65]. Both H-SELEX and transcriptome profiling point to RDTT regulating *dprA* expression (Fig. 6C). Importantly, *dprA* lacks an independent promoter and is thought to be transcribed from the upstream *sucCD* operon promoter, particularly during development of the competent state [21, 66, 67]. Transcriptome analyses suggest the presence of a Rho-stimulated IT terminator insulating *dprA* from *sucCD* transcription (Fig. 6C, pink symbol) [26]. Consistently, Δ*rho* cells exhibited strong *dprA* upregulation during exponential growth [21, 23], likely due to transcriptional read-through beyond the putative IT terminator (Fig. 6C). Northern blot analysis with an oligonucleotide probe targeting the upstream region of *dprA* revealed the presence of long (>3000 nt) transcripts in the Δ*rho* mutant (Fig. 6D), with sizes consistent with transcription initiating at the *sucC* promoter and extending to the end of the *dprA* gene (+3074 from start site) and beyond. Shorter transcripts (migrating as the 2928-nt 23S RNA) were detected in the Δ*pnp* strain but not in WT cells, likely due to their rapid degradation by PNPase. In the Δ*pnp* Δ*rho* strain, these shorter transcripts are replaced by longer RNA species, confirming their origin as RDTT products. Their length align with RDTT occurring at the *Rut* sites identified by H-SELEX (Fig. 6C).

To further investigate this mechanism, we performed live cell array experiments with *dprA*∼*gfp* reporter strains, in which the *gfp* gene is fused downstream of *dprA*, enabling both co-transcription and co-translation of the two genes (see “Materials and methods” section). Only background fluorescence was detected in WT cells carrying the dprA∼*gfp* fusion whereas substantial GFP fluorescence was detected in the Δ*rho* reporter strain (Fig. 6E), further supporting the role of RDTT in *dprA* regulation. To test whether transcriptional read-through from *sucCD* directly drives *dprA* expression, we inserted three strong IT terminators (λ phage’s *t_0_* and *E. coli*’s *rrnB t_1_*and *t_2_* terminators, which are highly effective in *B. subtilis*; [37]) just downstream of *sucD* in the Δ*rho* strain carrying the dprA∼*gfp* fusion. This insertion led to a dramatic decrease of GFP fluorescence (Fig. 6E), unambiguously demonstrating that *dprA* expression originates from upstream *sucCD* transcriptional read-through. Remarkably, inserting these insulating terminators also strongly decreased the DNA transformation efficiency of competent WT and Δ*rho* cells to levels comparable to a Δ*dprA* mutant (Fig. 6F). The absence of a significant difference in DNA transformation between WT and Δ*rho* cells (Fig. 6F) can be explained by the timing of DNA competence, which arises late in growth when Rho levels are low [23]. At this stage, WT cells likely produce enough DprA—through transcriptional read-through of the RDTT terminator—to perform its role in protecting and processing incoming DNA [68]. As a result, a difference in DprA levels between WT and Δ*rho* cells would not be detected by our assay, which measures the rate of chromosomal transformation.

Collectively, these results strongly support that RDTT initiated at intragenic *Rut* sites in *dprA* (Fig. 6C) generates Rho-dependent *sucCD-dprA* transcripts, which are rapidly processed by PNPase up to the position of the putative IT terminator. Whether this IT terminator functions as an active termination signal or merely serves as a steric block (Δ*G*_Mfold_ = −18.4 kcal/mol) to PNPase progression remains to be determined. In any case, these findings implicate both RDTT and RNA decay in the regulation of *dprA* expression and *B. subtilis* competence, further demonstrating that exoribonucleases can extensively process Rho-dependent transcripts, as previously described for *E. coli* [27, 28] and, to a lesser extent, for *B. subtilis* [69].

### H-SELEX mapping highlights Rho involvement in regulatory cascades

H-SELEX mapping can help identify indirect RDTT effects arising from regulatory cascades controlled by Rho-dependent regulators. For example, we identified putative *Rut* sites in 52 genes classified in the *Subti*Wiki functional category “Regulation of gene expression,” including sigma factor genes and transcription factor-encoding genes (Supplementary Table S7). This finding suggests that a substantial portion of the Rho-dependent effects observed in transcriptome and Term-seq profiling of Δ*rho* vs. WT cells may actually be indirect.

**Figure 7.**
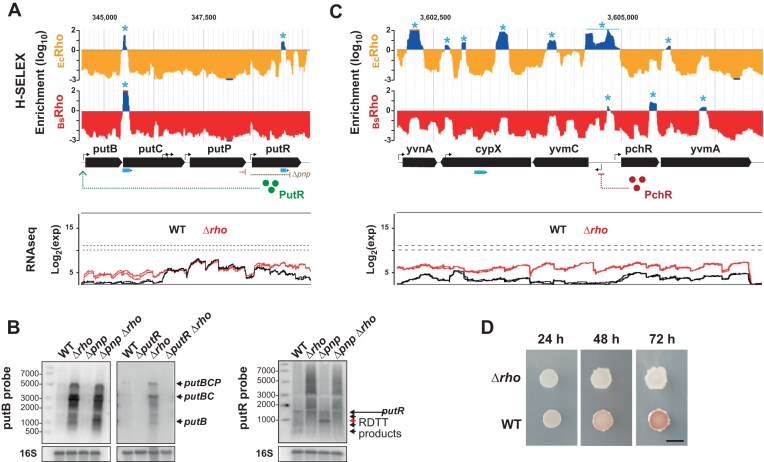
Indirect Rho-dependent regulation of the *putBCD* and *yvmC-cypX* operons. (**A**and **C**) H-SELEX enrichment (top) and RNAseq (bottom) profiles for the genomic regions of interest. Blue asterisks mark validated *Rut* peaks. The brown symbol shows the position of the *putR* transcript 3′-ends detected by northern blot in the Δ*pnp* strain. (**B**) Northern blot analysis of the *putBCD* and *putR* transcripts formed in the WT, Δ*pnp*, Δ*rho*, Δ*pnp* Δ*rho*, Δ*putR*, and *Δrho ΔputR* strains using riboprobes targeting either *putB* (left) or *putR* (right). (**D**) Deletion of *rho* reduces pulcherrimin production. Five microliters of overnight cultures of either *B. subtilis* BsB1 (WT) or an isogenic Δ*rho* strain were spotted onto MSgg agar plates containing 50 μM FeCl_3_ and incubated at 30°C. Images were captured at the specified incubation times. Pulcherrimin production was assessed based on the development of the red pigment associated with the insoluble pulcherrimin–iron complex [35]. The black scale bar corresponds to 5 mm.

A representative example is the *putBCPR* locus involved in proline utilization, which contains *Rut* peaks in the upstream section of *putC* and the downstream section of *putR* (Fig. 7A). The *putR* gene encodes a transcription factor that positively regulates the *putBCP* operon and is transcribed from its own promoter, with at least partial insulation from upstream *putBCP* transcription by an IT terminator (Fig. 7A) [21, 26]. To investigate *putBCP* expression, we performed northern blot analysis using an oligonucleotide probe targeting *putB*. We observed a high increase of the level of *putBCP* transcripts in the Δ*rho* background (Fig. 7B, left), confirming Rho’s involvement in regulating the expression of this operon. Surprisingly, PNPase inactivation had little impact on these results: *putBCP* transcripts remained barely detectable in the Δ*pnp* strain while their distribution in the double mutant Δ*pnp* Δ*rho* closely resembled that in the Δ*rho* strain (Fig. 7B, left). Two distinct scenarios could explain these results: (i) unstable Rho-dependent transcripts—originating from the *Rut* site in *putC* (Fig. 7A)—are rapidly degraded in a PNPase-independent manner in the WT and Δ*pnp* strains, or (ii) the formation of *putBCP* transcripts in the Δ*rho* background primarily results from the upregulation of *putR* following the bypass of its intragenic *Rut* site. Several lines of evidence strongly support the second scenario. First, we could no longer detect *putBCP* transcripts in the Δ*rho* background when *putR* was also deleted (Fig. 7B, Δ*putR* Δ*rho* strain), confirming that PutR is strictly required for *putBCP* expression. Second, northern blot analysis using a *putR*-specific probe revealed that *putR* transcripts are largely truncated in the WT strain (Fig. 7B, right). In the Δ*pnp* strain, truncated *putR* transcripts are less diffusely distributed, and the predominant species (∼1000 nt, red arrow) is consistent with RDTT originating from the intragenic *Rut* site detected in *putR* by H-SELEX (Fig. 7A). Furthermore, the disappearance of truncated transcripts in favor of longer RNA species in the Δ*rho* background (Fig. 7B, right) supports direct Rho-dependent regulation of *putR* expression. Finally, independent transcriptome and proteome analyses have confirmed the increased expression of the *putR* gene and PutR protein in the Δ*rho* strain [22]. Thus, RDTT directly regulates PutR abundance, which in turn controls *putBCP* transcription (Fig. 7A).

Another mechanism of indirect RDTT regulation arises when a gene encoding a regulatory protein (or ncRNA) is insulated from the upstream transcription units by Rho-dependent terminator(s). In such cases, transcription read-through through the terminator(s) can activate regulatory cascades controlled by the affected regulator. For instance, several putative *Rut* sites were identified in the gene cluster containing the divergently transcribed gene pairs *pchR-yvmA* and *yvmC*-*cypX* (Fig. 7C), which are involved in the biosynthesis of the iron chelator pulcherrimin [70]. Transcription through these *Rut* sites when Rho levels are low would enhance *pchR* expression, leading to increased levels of PchR, a transcriptional repressor of the *yvmC*-*cypX* locus and an activator of *yisI* [70]. Comparative transcriptome and proteome analyses support this model, showing that *pchR* expression increases ∼5-fold in Δ*rho* cells—primarily due to transcription read-through from adjacent upstream genes (Fig. 7C)—while *yisI* is upregulated ∼8-fold and *yvmC* and *cypX* are downregulated ∼7-fold [22, 23]. This dysregulation drastically reduces the production of the red-colored pulcherrimin-iron complex in the Δ*rho* mutant (Fig. 7D).

## Conclusions

H-SELEX is a high-throughput approach that enabled the precise mapping of putative *Rut* sites utilized by the Rho helicase across the *E. coli* genome [10]. H-SELEX revealed Rho-dependent regulatory signals that were overlooked in standard transcription profiling analyses [29], demonstrating its value in charting the RDTT landscape. Using a similar experimental strategy, we now provide an extensive, high-resolution perspective of RDTT in *B. subtilis*, independent of growth conditions, transcriptional signal strength, or indirect regulatory effects.

Notably, H-SELEX reveals that *in vivo* RDTT transcript 3′-ends are frequently located upstream from the nearest *Rut* site (Fig. 4E). This observation aligns with the reported enrichment of *Rut*-like features—C > G “bubble” density and YC repeats—downstream rather than upstream of these 3′-ends [26]. These findings highlight the extensive post-transcriptional processing of Rho-dependent transcripts and underscore the challenges in mapping RDTT sites precisely using transcription profiling or Term-seq approaches alone.

Using northern blot analysis, we show that PNPase plays a major role in processing representative RDTT transcripts in *B. subtilis* (Figs 6B, D and 7B, right). Previous work showed that *slrA* transcripts similarly undergo RDTT followed by PNPase processing, though to a substantially lesser extent [69], underscoring variability in PNPase-mediated transcript processing in *B. subtilis*.

Our H-SELEX map (Supplementary Table S4) reveals an elaborate Rho-dependent regulatory network, influencing both primary *(*e.g. Figs 6A and 7A) and secondary (e.g. Fig. 7C and D) metabolism, in connection with *B. subtilis* adaptive programs. While putative *Rut* sites in the antisense orientation are prevalent (Fig. 4B)—consistent with transcription profiling analyses (Supplementary Fig. S8) [21, 22]—we also identified functionally significant examples of sense-oriented RDTT terminators (Figs 5 and 6). Although these various RDTT signals are dispensable for *B. subtilis* growth under standard laboratory conditions [71], they likely play a more critical role in bacterial fitness—beyond sporulation [23, 25]—under the challenging conditions encountered in natural environments.

**Figure 8.**
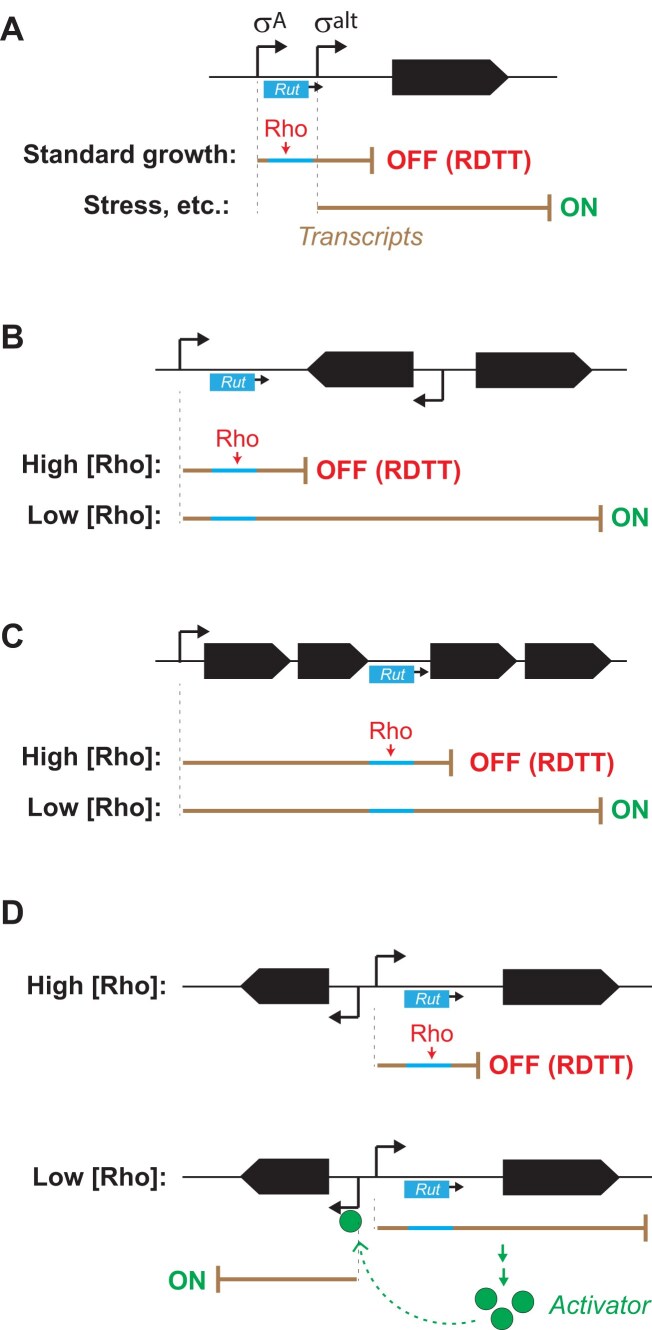
Models of conditional RDTT. (**A**) Regulation by alternative promoter and Sigma factor. (**B–** **D**) Regulation by terminator read-through at low cellular Rho concentration. This may lead to (B and C) direct or (D) indirect expression of the gene(s) of interest. The case where expression of a repressor rather than activator is controlled by Rho level is also possible but not shown.

Our findings suggest several models for conditional RDTT in *B. subtilis*, which may extend to other bacteria. First, promoter-proximal *Rut* sites (Fig. 4D), especially those near promoters driven by alternative Sigma factors (Supplementary Table S4), may represent RDTT signals that are either active or silent depending on specific stress or growth conditions (Fig. 8A). Second, transcription through “leaky” RDTT signals could regulate downstream genes (Fig. 8B and C). In this model, terminator leakiness may depend on specific cofactors or intracellular Rho concentration, which fluctuates depending on growth stage in *B. subtilis* [21, 23–25] and, possibly, in other contexts. Lastly, conditional RDTT may indirectly control regulatory cascades (Fig. 8D), as seen in the *putBCPR* locus where RDTT modulates expression of PutR (Fig. 7A), the transcriptional activator of *putBCP* [72]. These regulatory dynamics could facilitate adaptive shifts in gene expression.

Additionally, conditional RDTT in long biosynthetic operons (Fig. 8C) may help fine-tune metabolic fluxes in *B. subtilis* cells, as observed for the *pks* and *alb* operons (see Fig. 5 and “Results” section) [23]. Interestingly, the *albE* and *albF* genes—encoding proteins required for the final maturation of the subtilosin antibiotic [73]—are located downstream in the *albABCDEFG* operon and are downregulated by Rho from a *Rut* site in *albF* (Supplementary Table S4). This regulatory arrangement may ensure that the biosynthesis of subtilosin is distributed over the course of the growth cycle, with the final maturation step occurring predominantly during the stationary phase, when Rho levels decrease and *albE/F* are more efficiently transcribed [21, 23, 24]. The Rho-dependent, delayed maturation of this secondary metabolite may be linked to quorum sensing and the more pressing need to eliminate competitors in densely populated environments.

Previous studies indicate that NusA and NusG influence the biogenesis of Rho-dependent transcripts in *B. subtilis*, with NusA playing the most significant role [26]. Regulation of _Bs_Rho’s activity by cofactors and *cis-*acting RNA sequences may contribute to compensate for the weak coupling between transcription and translation in *B. subtilis*, a condition that could otherwise result in spurious RDTT within CDSs [63]. Our findings that _Bs_Rho has weaker enzymatic activity (Figs. 2D and Supplementary S2C) and slightly more constrained *Rut* site preferences (see “Results” section) than _Ec_Rho support this hypothesis. Whether NusA, NusG, or other factors fine-tune RDTT beyond a purely constitutive process remains an open question, offering another exciting direction for future research.

### Limitations of the study

Unlike standard SELEX, H-SELEX selects RNAs based on their ability to activate Rho enzymatically, rather than solely on binding affinity [10]. As a result, the RNA sequences identified (putative *Rut* sites) represent *bona fide* substrates for the Rho enzyme. However, genome-wide identification of these sites relies on peak-calling algorithms, which are imperfect [74]. While we adopted a conservative strategy by cross-validating results from two independent peak callers, we cannot fully rule out the presence of false positives or negatives in our final list of putative *Rut* sites (Supplementary Table S4). Moreover, as an *in vitro* approach, H-SELEX may identify *Rut* sequences that are rarely or never engaged by Rho *in vivo*, either because they are not transcribed or are occluded by other RNA-binding factors.

The resolution of *Rut* site boundaries deduced by H-SELEX is inherently limited by the variable lengths of the gDNA fragments in the initial R_0_ library, which are generated by random priming and PCR amplification of gDNA. Some fragments may exceed the actual size of the *Rut* sites, leading to enrichment of longer transcripts and, consequently, to *Rut* peaks that are broader than the functional motifs they encompass (Supplementary Fig. S1C) [10].

## Supplementary Material

gkaf765_Supplemental_Files

## Data Availability

The raw NGS data for the H-SELEX libraries have been deposited in the European Nucleotide Archive under accession code PRJEB88389. The H-SELEX enrichment profiles can be visualized alongside transcriptome profiles from ref. [23] on the Genoscapist website at https://genoscapist.migale.inrae.fr/seb_rut/.
