# Supplementary material for: Comprehensive mapping of transcription terminator Rho utilization (Rut) sites across the Bacillus subtilis genome
Source: Nucleic Acids Res. 2025 Aug 13;53(15):gkaf765. doi: 10.1093/nar/gkaf765 (PMC12350095; doi:10.1093/nar/gkaf765)
Supplement: gkaf765_Supplemental_Files [file gkaf765_supplemental_files.zip › H-SELEX_BsRho supplementary info_NAR_revised_v4.pdf]

Comprehensive mapping of transcription terminator *R*ho *ut*ilization (*Rut*) sites across the *Bacillus subtilis* genome

Mildred Delaleau[1,†], Vladimir Bidnenko[2,†], Eric Eveno[1,†], Gergana Kostova[3], Johnathan C. Black[1,4], Stephen McGovern[2], Olivier Pellegrini[3], Sandra Dérozier[4], Matthieu Jules[2], Ciaran Condon[3], Sylvain Durand[3,*], Elena Bidnenko[2,*], and Marc Boudvillain[1,5,*]

(Supplementary information: Tables S1 to S3 and Figures S1 to S11)

Table S1 : Oligonucleotides (sequences are 5'→3')

R45-ran-Rev	AGATCTGATACCATCGNNNNNNNNN
ARN107-ran-For	GGGAGACCGGCCAGCNNNNNNNNN
FWD	CGAAATTAATACGACTCACTATAGGGAGACCGGCCAGC
REV	CGATGAATTCGAGCTCGGTACCCGCAGCTTCTCGAGGATCCAGATCTGATACCATCG
SEL	Biotin~TTTTTTTTTTCGATGAATTCGAGCTCGGTACCCGCAGCTTCTCGAGGATCCAGATCTGATACCATCG
BLOCK	GCTGGCCGGTCTCCC
TRAP	CGATGGTATCAGATCTGGATCCTCGAGAAGCTGCGGGTACCGAGCTCGAATTCATCG
IRUT-OLN	GGGAGACCGGCCAGCGGTCGAGGAAGAAGGAGACAGAGGAGAAGGAAGAAGGAGACAGAGGAGAAGTCGATGGTATCAGATCTGG
LESS-OLN	GGGAGACCGGCCAGCCGATGGTATCAGATCTGG
FORWA	CGGCTCGTATAATGTGTGGAATTGTGAGCGGATAACAATTTGGGAGACCGGCCAGC
FORWB	GCGGCCGCACTCGAGGAGCTGTTGACAATTAATCATCGGCTCGTATAATGTGT
FACS-REV	ACTGTCTTACACACCGGTAAGACAGCCCAGATCTGATACCATCG
C1-OLN	GGGAGACCGGCCAGCCCCATGTATCGTCGAGGGCAGTTCTTGGATCCTCTGTAAGAGATTACGGTTATCTCCGTATGAAACAGTTGTTTACCCTGCGATGGTATCAGATCTGG
citZ riboprobe Fwd. (CC2166)	CAAAGGGTTTTAGTTCCAGCAGC
citZ riboprobe Rev. (CC2167)	GCTCTAATACGACTCACTATAGGGATACCCCACATATGTAAGGGTATCATC
putR riboprobe Fwd. (CC3526)	CAGTGATACTGGAAAGCGCGG
putR riboprobe Rev. (CC3527)	GCTCTAATACGACTCACTATAGGGTCCAGCTCCTGAATCCAG
putB riboprobe fwd. (CC2719)	GAGGACGAAGTCAGATGCCAG
putB riboprobe Rev. (CC2720)	GCTCTAATACGACTCACTATAGGACCGCATGCCGTACAGC
dprA probe (CC3518)	GGCAGACAGCGGCCTGATCC
veb734	CTAGACCCGGGGATCTCTG
veb735	TCTAGAGCAACGTTCTTGC
veb876 *	AAGAACGTTGCTCTAGAAAAAAAGGGACAGCCGTCAA
veb946	ACGGATCCTCATAGGGAAATG
veb867	GAAGAATTCAAACAACCGGC
veb947 **	GAGATCCCCGGGTCTAGATTAATGCGTTTTACAAGTTTCG
veb977 ***	TCCAATAGCCGTCATAAATATAAAAAAAGAAGG
veb978 ***	TATATTTATGACGGCTATTGGACCATCTAGATGTGGGAGC
veb976	AACGTCGACTGAAGTTACAATGG
veb979	CACAGGATCCAGCTGGCAAAG
veb740	ATGAGTAAAGGAGAAGAAC
veb741	ACGCGTCGACTTATTTGTATAGTTCATCCATG
veb872	GTGGAATTCCGATCCGTCTATGTC
veb874 ****	*GTTCTTCTCCTTTACTCAT*AAGGGTTCCGTATATTGAACG
veb982 *****	TTCTTCTCCTTTTGACATGTGTATAACCTTCTTTTTTTA
veb983	TACGAATTCATGAAACAGAC
veb989	CGAGGATCCAGAATGAAGATTTAC
veb990	GCTGTCGACGAGCCATTTATAAAAG
veb991	ATGAGTCTTGTGGAAGTATG
veb992	GAGTTAGCTCACTCATTAGG

(*) sequence complementary to veb735 is underlined
(**) sequence complementary to veb734 is underlined
(***) complementary sequences are underlined
(****) sequence complementary to veb740 is in italics; overlapping stop codon of *dprA* and start codon of *gfp* genes are underlined
(*****) sequence complementary to veb740 is underlined

**Table S2 : Bacterial strains and plasmids**		
**Strain**	**Relevant genotype (drug resistance)**	**Source or reference**
BsB1	*B.subtilis* 168 trp^+	[1]
NCIB3610	*B.subtilis* prototroph Marburg	[2]
BRL1	BsB1 *Δrho::phleo* (Pm^R)	[3]
BRL1412	BsB1 *sucD-IT*	This study
BRL1477	BsB1 *Δrho sucD-IT* (Pm^R)	This study
BRL1296	BsB1 *dprA~gfp* (Sp^R)	This study
BRL1297	BsB1 *Δrho dprA~gfp* (Pm^R Sp^R)	This study
BRL1414	BsB1 *sucD-IT dprA~gfp*	This study
BRL1454	BsB1 ΔRut^{pks}	This study
BRL1487	BsB1 *pksDΩgfp* (Sp^R)	This study
BRL1488	BsB1 ΔRut^{pks} *pksDΩgfp* (Sp^R)	This study
BRL1489	BsB1 *Δrho pksDΩgfp* (Pm^R ; Sp^R)	This study
BRL1455	BsB1 sfp^+ (Sp^R)	This study
BRL1458	BsB1 *Δrho* sfp^+ (Pm^R ; Sp^R)	This study
BRL1461	BsB1 ΔRut_{pks} sfp^+ (Sp^R)	This study
BKK17140	BsB1 *ΔpksF* (Km^R)	[4]
BRL1480	BsB1 sfp^+ *ΔpksF* (Sp^R Km^R)	This study
BRL1481	BsB1 *ΔRho* sfp^+ *ΔpksF* (Pm^R ; Sp^R ; Km^R)	This study
CCB1922	BsB1 *Δpnp::Cm* (Cm^R)	This study
CCB1923	BsB1 *Δrho::phleo Δpnp::Cm* (Pm^R ; Cm^R)	This study
CCB1966	*Bsb1 ΔputR::cm* (Cm^R)	This study
CCB1967	*Bsb1 Δrho::phleo ΔputR::cm* (Pm^R ; Cm^R)	This study
1884	*Bacillus thuringiensis* sv. *israelensis 1884*	Nielsen-LeRoux (Micalis, INRAE)
KBAB4	*Bacillus weihenstephanensis* KBAB4	Nielsen-LeRoux (Micalis, INRAE)
**Plasmid**	**Drug resistance (*B. subtilis*/*E. coli*)**	**Source or reference**
pMutin4	Em^R/Ap^R	[5]
pMAD	Em^R/Ap^R	[6]
pCVO119	Sp^R/Ap^R	[7]
pCVO119m	pCVO119(*gfpA206K*); Sp^R/Ap^R	A. Chastanet (Micalis, INRAE)
pBRL892	pCVO119 Δ*gfp*; Sp^R/Ap^R	This study
pBRL1408	pMAD with *sucD-IT* fragment; used to construct BRL1412; Em^R/Ap^R	This study
pBRL1450	pMAD with ΔRut_{pks} fragment; used to construct BRL1454; Em^R/Ap^R	This study
pBRL1292	pBRL892 with *dprA~gfp* fragment; used to construct BRL1297 and BRL1414; Sp^R/Ap^R	This study
pBRL1484	pBRL892 with *pksDΩgfp* fragment; used to construct BRL1487; Sp^R/Ap^R	This study
pBRL1485	pBRL892 with ΔRut_{pks} *pksDΩgfp* fragment; used to construct BRL1488; Sp^R/Ap^R	This study
pBRL1452	pBRL892 with spf^+ gene from NCBI3610; used to construct BRL1455, BRL1458 and BRL1461; Sp^R/Ap^R	This study
pET28a-BsRho	Derivative of commercial pET28a plasmid, used for $_{Bs}$Rho overexpression	This study

Table S3: Duration of duplex unwinding reactions during H-SELEX rounds (final libraries are in blue)			
**Round**	**Selection time (s)**	**Libraries obtained with $_{Bs}$Rho**	**Libraries obtained with $_{Ec}$Rho**
1	600	$_{Bs}R_{1a,b}$	$_{Ec}R_{1a,b}$
2	300	$_{Bs}R_{2a,b}$	$_{Ec}R_{2a,b}$
3	150	$_{Bs}R_{3a,b}$	$_{Ec}R_{3a,b}$
4	120	$_{Bs}R_{4a,b}$	$_{Ec}R_{4a,b}$
5	120	$_{Bs}R_{5a,b}$	$_{Ec}R_{5a,b}$
6	60	$_{Bs}R_{6a,b}$	$_{Ec}R_{6a,b}$
7	30	$_{Bs}R_{7a,b}$	$_{Ec}R_{7a,b}$
8	20	$_{Bs}R_{8a,b}$	$_{Ec}R_{8a,b}$
9	20	$_{Bs}R_{9a,b}$	$_{Ec}R_{9a,b}$
10	20	$_{Bs}R_{10a,b}$	$_{Ec}R_{10a,b}$
11	20	$_{Bs}R_{11a,b}$	x
12	20	$_{Bs}R_{12a,b}$	x
13	20	$_{Bs}R_{13a,b}$	x
14	20	$_{Bs}R_{14a,b}$	x

REFERENCES FOR SUPPLEMENTARY TABLES

1. Nicolas, P., Mader, U., Dervyn, E., Rochat, T., Leduc, A., Pigeonneau, N., Bidnenko, E., Marchadier, E., Hoebeke, M., Aymerich, S. *et al.* (2012) Condition-dependent transcriptome reveals high-level regulatory architecture in Bacillus subtilis. *Science*, **335**, 1103-1106.
2. Zeigler, D.R., Pragai, Z., Rodriguez, S., Chevreux, B., Muffler, A., Albert, T., Bai, R., Wyss, M. and Perkins, J.B. (2008) The origins of 168, W23, and other Bacillus subtilis legacy strains. *J Bacteriol*, **190**, 6983-6995.
3. Bidnenko, V., Nicolas, P., Grylak-Mielnicka, A., Delumeau, O., Auger, S., Aucouturier, A., Guerin, C., Repoila, F., Bardowski, J., Aymerich, S. *et al.* (2017) Termination factor Rho: From the control of pervasive transcription to cell fate determination in Bacillus subtilis. *PLoS Genet*, **13**, e1006909.
4. Koo, B.M., Kritikos, G., Farelli, J.D., Todor, H., Tong, K., Kimsey, H., Wapinski, I., Galardini, M., Cabal, A., Peters, J.M. *et al.* (2017) Construction and Analysis of Two Genome-Scale Deletion Libraries for Bacillus subtilis. *Cell systems*, **4**, 291-305 e297.
5. Vagner, V., Dervyn, E. and Ehrlich, S.D. (1998) A vector for systematic gene inactivation in Bacillus subtilis. *Microbiology (Reading)*, **144 (Pt 11)**, 3097-3104.
6. Arnaud, M., Chastanet, A. and Debarbouille, M. (2004) New vector for efficient allelic replacement in naturally nontransformable, low-GC-content, gram-positive bacteria. *Appl Environ Microbiol*, **70**, 6887-6891.
7. Eichenberger, P., Jensen, S.T., Conlon, E.M., van Ooij, C., Silvaggi, J., Gonzalez-Pastor, J.E., Fujita, M., Ben-Yehuda, S., Stragier, P., Liu, J.S. *et al.* (2003) The sigmaE regulon and the identification of additional sporulation genes in Bacillus subtilis. *J Mol Biol*, **327**, 945-972.

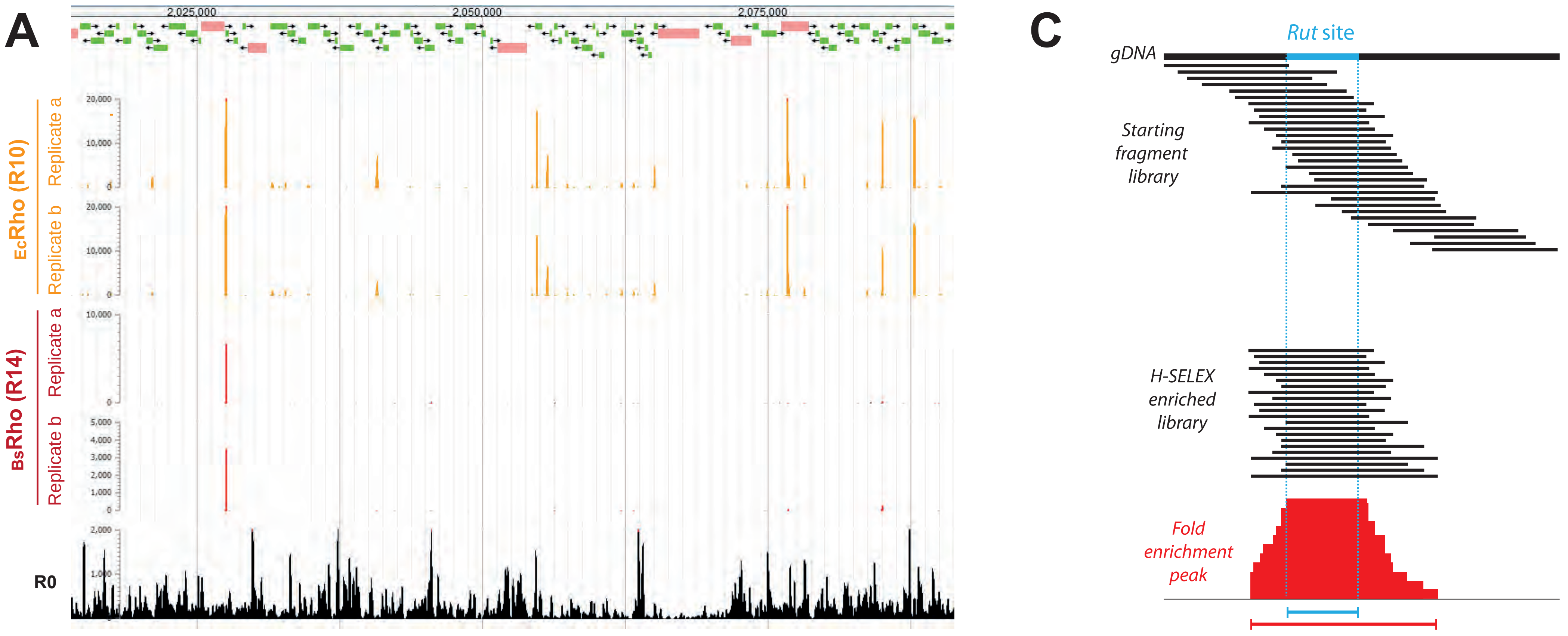

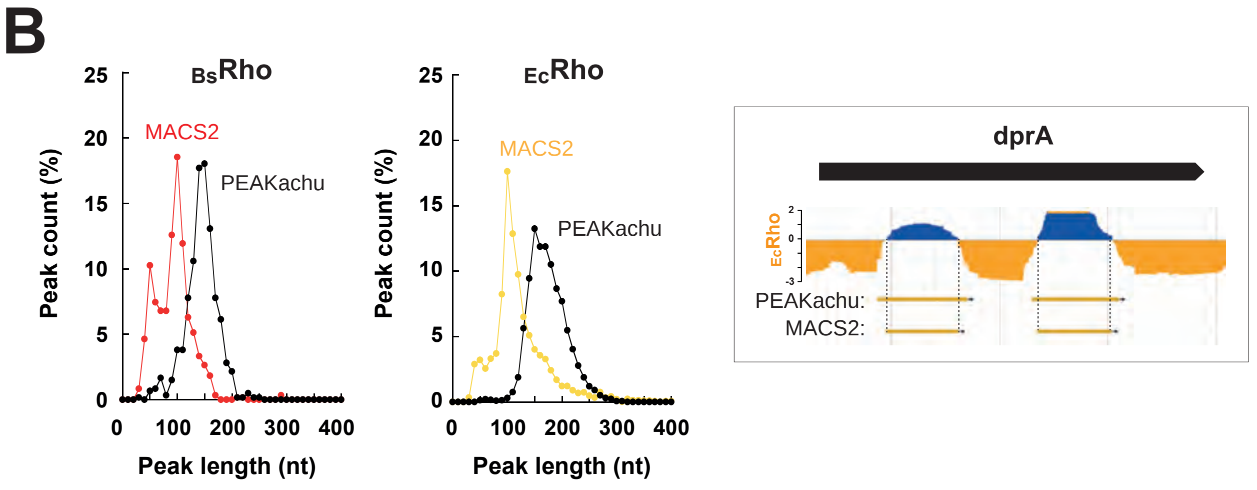

Supplementary Figure S1: (A) Normalized read coverages for a representative genomic region (minus strand) illustrate the >98% genome coverage achieved by the starting R_0 library and the high reproducibility between H-SELEX-enriched library duplicates. **(B)** Length distributions of *Rut* peaks identified using the MACS2 and PEAKachu algorithms. MACS2 consistently yielded narrower peaks than the corresponding PEAKachu calls, and better defined the boundaries of the $Log_{10}FE$ enrichment peaks, as shown in the inset example. This likely reflects an inherent limitation of PEAKachu's adaptive detection mode. **(C)** The precision in defining *Rut* site boundaries is influenced not only by the peak calling algorithm (as shown in panel B) but also by the size range of the gDNA fragments (and corresponding transcripts) in the starting library. Transcripts overlapping *Rut* sequences are selectively enriched by H-SELEX, resulting in enrichment peaks that may extend beyond the actual *Rut* site boundaries. The figure depicts sequencing reads after removal of the constant flanking sequences used in the H-SELEX protocol (Fig. 2A).

A

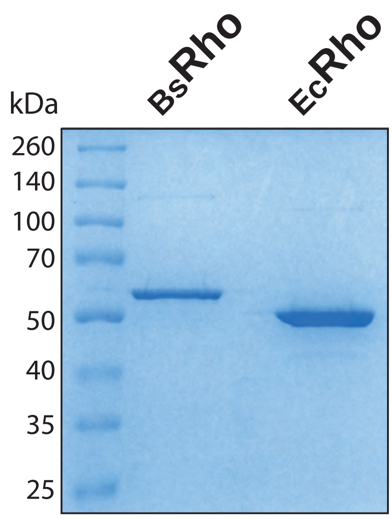

B

	ATPase activity (ATP/hexamer/s)	
$_{Ec}$Rho	111 ± 10	
$_{Bs}$Rho	40 ± 4	(native)
	10 ± 3	(renaturation)

C

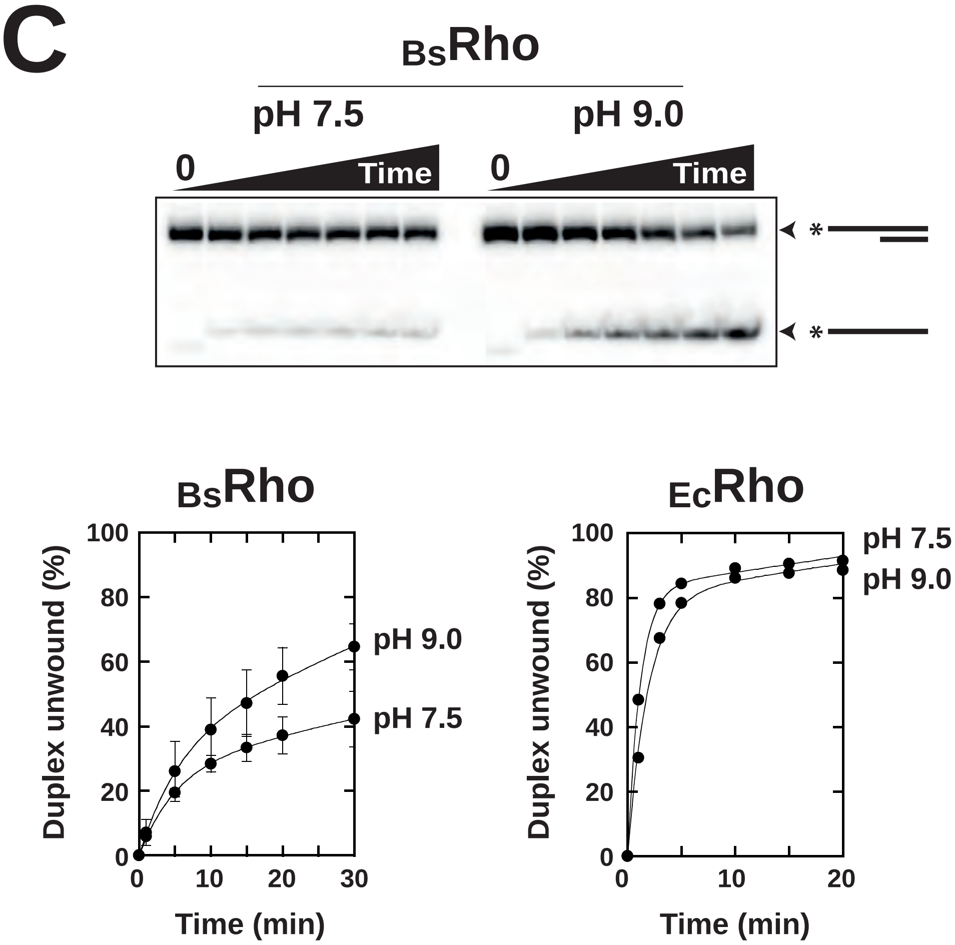

Supplementary Figure S2: Comparison of the purified $_{Bs}$Rho (50.43 kDa) and $_{Ec}$Rho (47.2 kDa) proteins. **(A)** Migration in a 10% SDS-PAGE gel. **(B)** Steady-state ATPase activity measured at 37°C in presence of excess poly(rC) in ATPase buffer (50 mM KCl, 1 mM $MgCl_2$, 0.1 mM DTT, 0.1 mM EDTA, 20 mM HEPES, pH 7.9). **(C)** Duplex unwinding activity measured at 30°C with 5nM of ^{32}P-labeled RNA-DNA hybrid (substrate C in Soares et al., 2014), 20 nM Rho, and 1 mM ATP in helicase buffer (150 mM Potassium Acetate, 100 nM $CaCl_2$, 10 mM NaCl, 0.1 mg/mL BSA and 25 mM HEPES pH 7.5 or 25 mM EPPS pH 9.0).

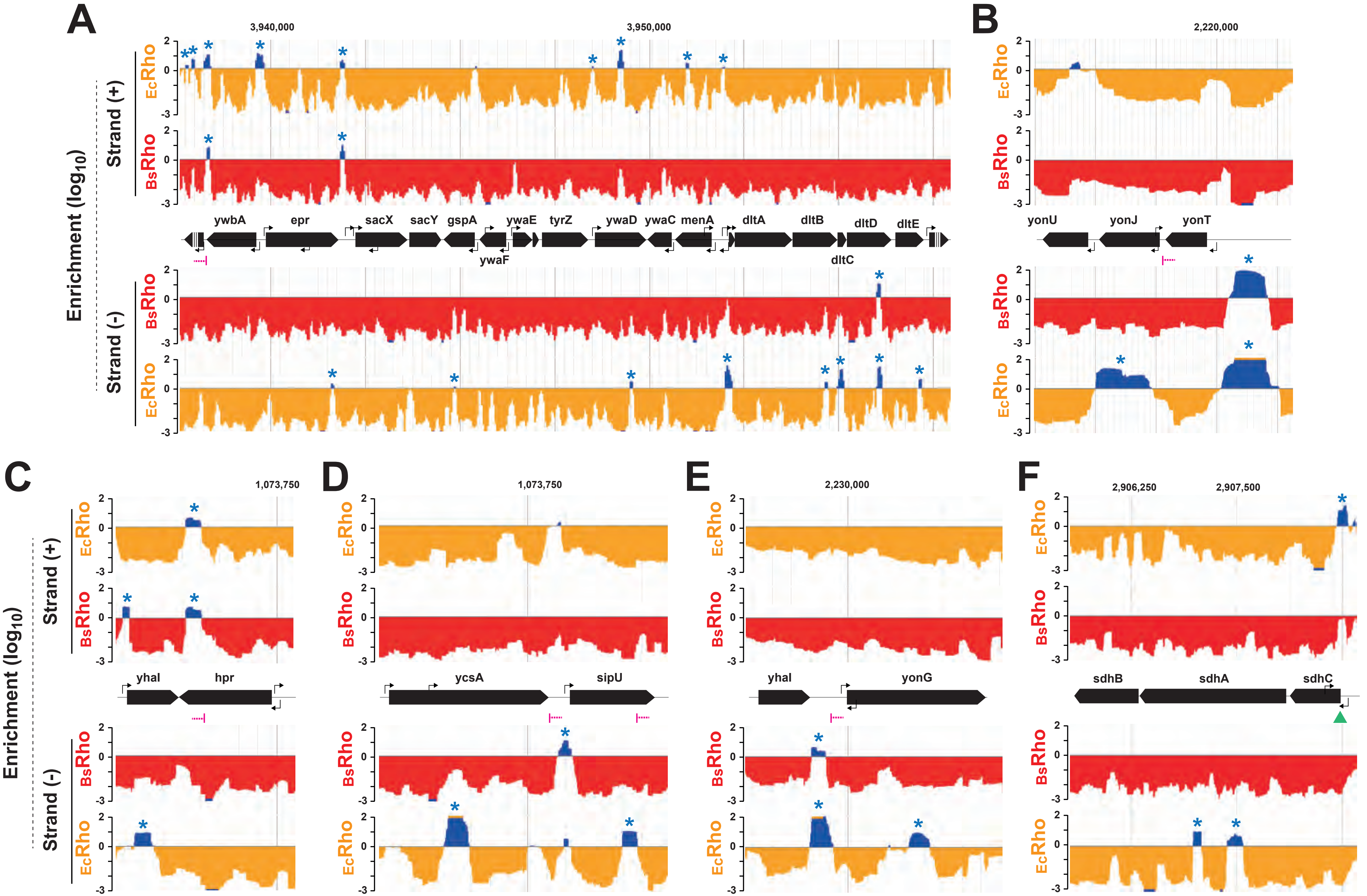

Supplementary Figure S3: Examples of enrichment profiles (Log_{10} scale) for selected regions of the *B. subtilis* genome (different scales along x-axis). Positive values are in dark blue while negative values are in orange ($_{Ec}$Rho) or red ($_{Bs}$Rho). Validated *Rut* peaks are marked by cyan asterisks. Pink symbols mark *in vivo* transcript 3'-ends assigned to RDTT (Mandell et al., 2022). The green triangle in panel F indicate the position of hybridization of sRNA FsrA.

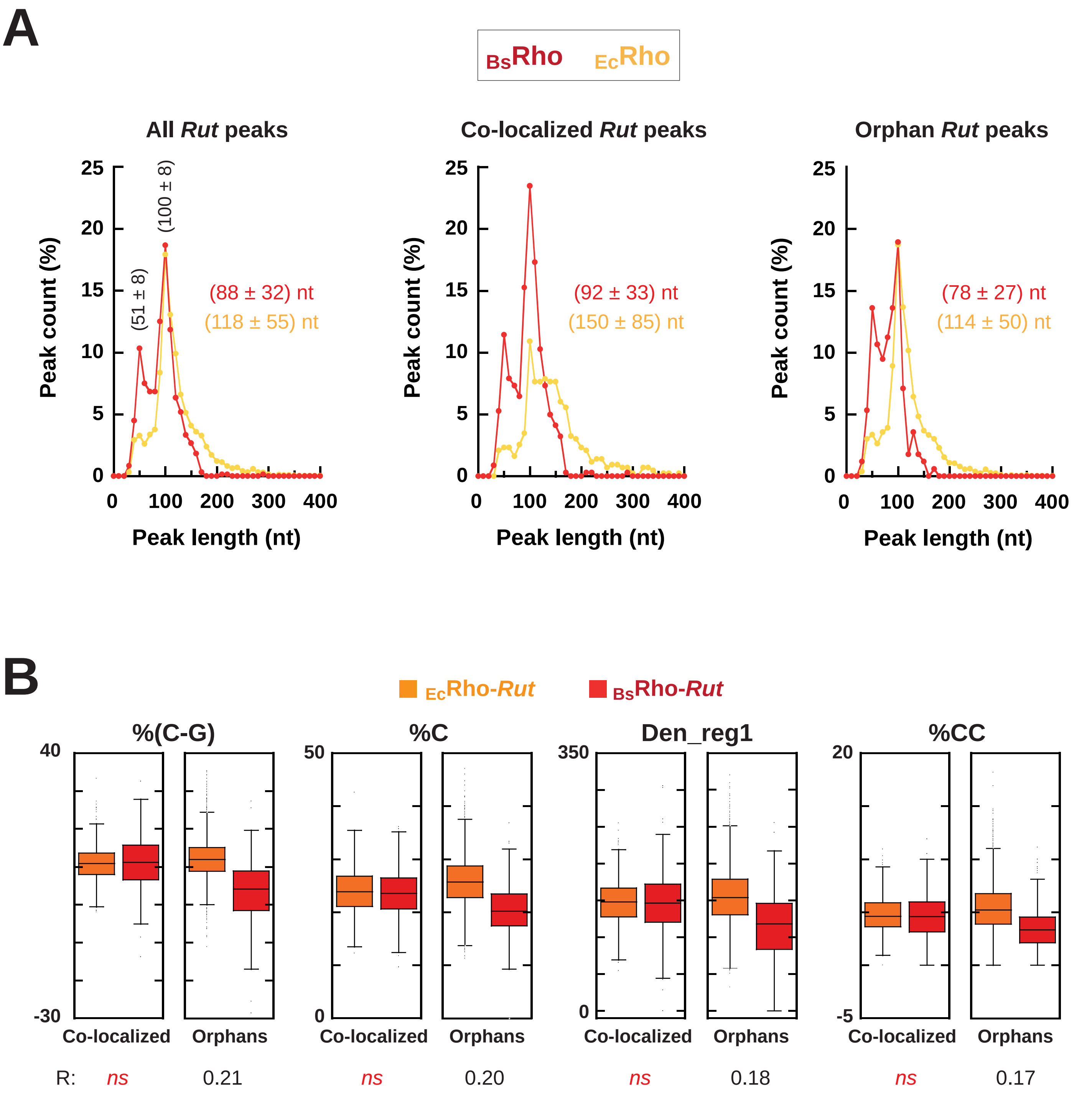

Supplementary Figure S4: (A) Distributions of the lengths of *Rut* peaks obtained with $_{Bs}$Rho and $_{Ec}$Rho (Figure 3C). Mean peak lengths (± SD) are indicated on each graph. On the left graph, (mean ± SD) lengths for the two subpopulations of peaks were estimated by fitting the $_{Bs}$Rho-*Rut* data to an equation describing a double Gaussian distribution. **(B)** Box plots for the four sequence descriptors that most discriminate orphan *Rut* peaks. Box plots for co-localized Rut peaks are shown for comparison. Effect sizes (R) for Mann-Whitney U tests comparing $_{Bs}$Rho-*Rut* (red) vs. $_{Ec}$Rho-*Rut* (orange) sequences are displayed below the box plots (*ns* if P-value > 0.05). Den-reg1 is the normalized number of 5’YC motifs (per kB), where Y is a pyrimidine residue.

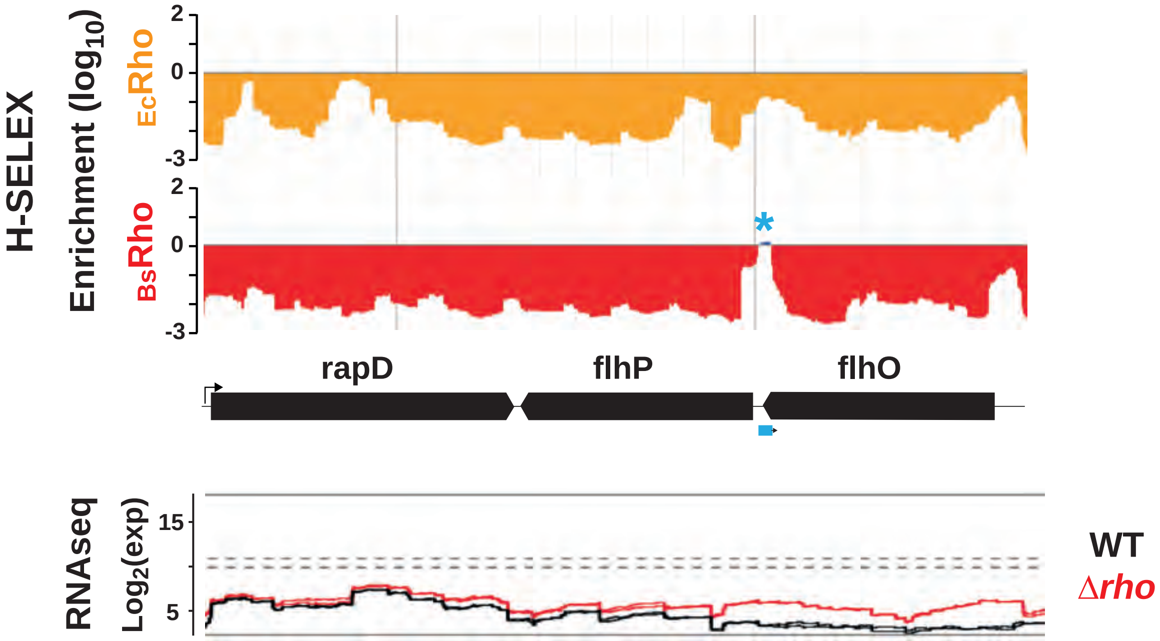

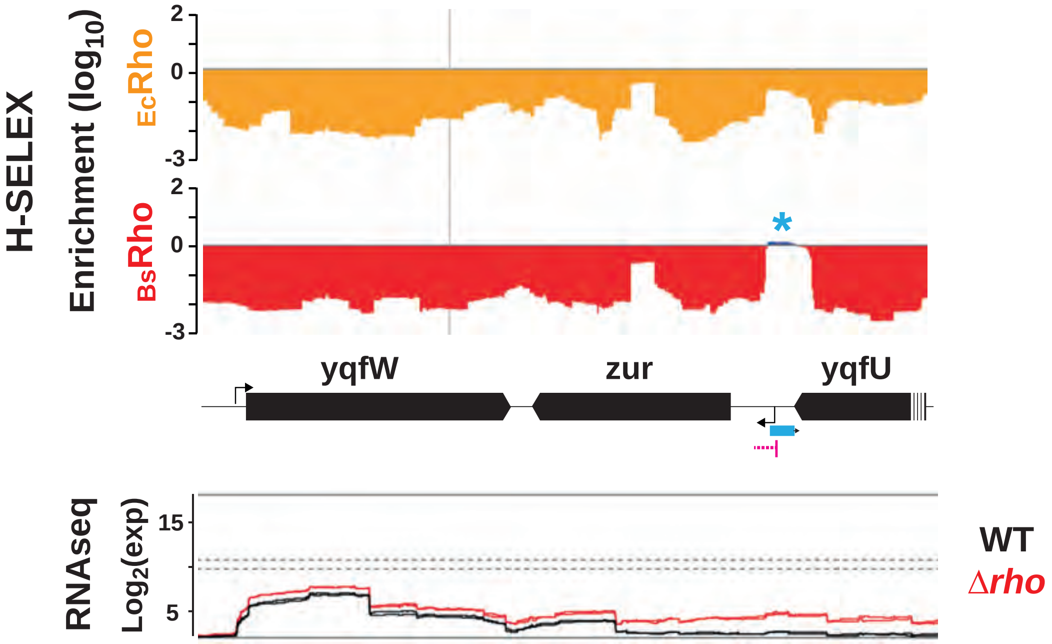

Supplementary Figure S5: Examples of short orphan $_{Bs}$Rho-*Rut* peaks that may have a functional role *in vivo*, as inferred from a comparison of H-SELEX enrichment (this work) and RNAseq (Bidnenko et al., 2023) profiles. In the H-SELEX enrichment profiles (Log_{10} scale), positive values are in dark blue while negative values are in orange ($_{Ec}$Rho) or red ($_{Bs}$Rho). Validated *Rut* peaks are marked by cyan asterisks and rectangles. RNAseq snapshots were taken from the genoscapist website (Derozier et al., 2021). In the case of the *flhOP* operon, we have shown previously that antisense transcription in the Δ*rho* mutant negatively affects motility (Bidnenko et al., 2017), thereby suggesting a regulatory role for the orphan *Rut* site detected in the (+) strand. In the case of the *yqfW* gene, a Rho-stimulated IT signal has been found farther downstream (pink symbol) (Mandell et al., 2022) , in close proximity to the orphan *Rut* peak.

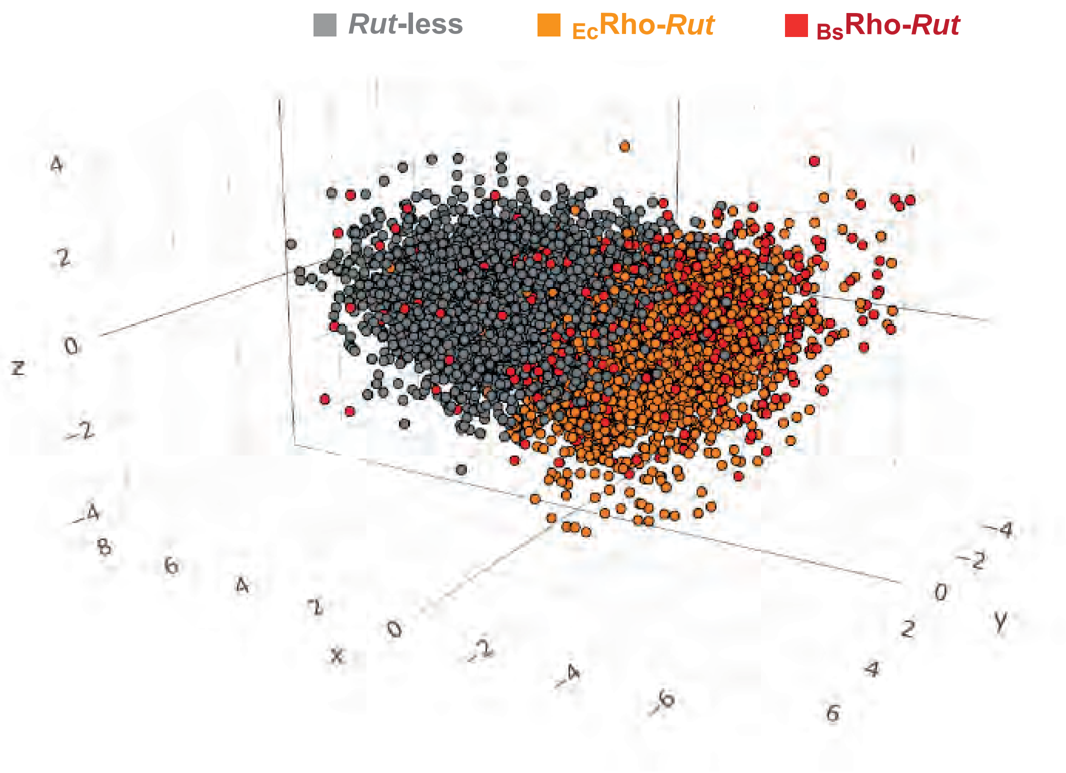

Supplementary Figure S6: Example of principal component analysis (PCA) performed with the most discriminative sequence descriptors (R > 0.3) obtained upon pairwise comparison of $_{Bs}$Rho-*Rut* and *Rut*-less sequences (see Table S3). As shown in this example, no combination of descriptors provided a good PCA separation among sequence categories.

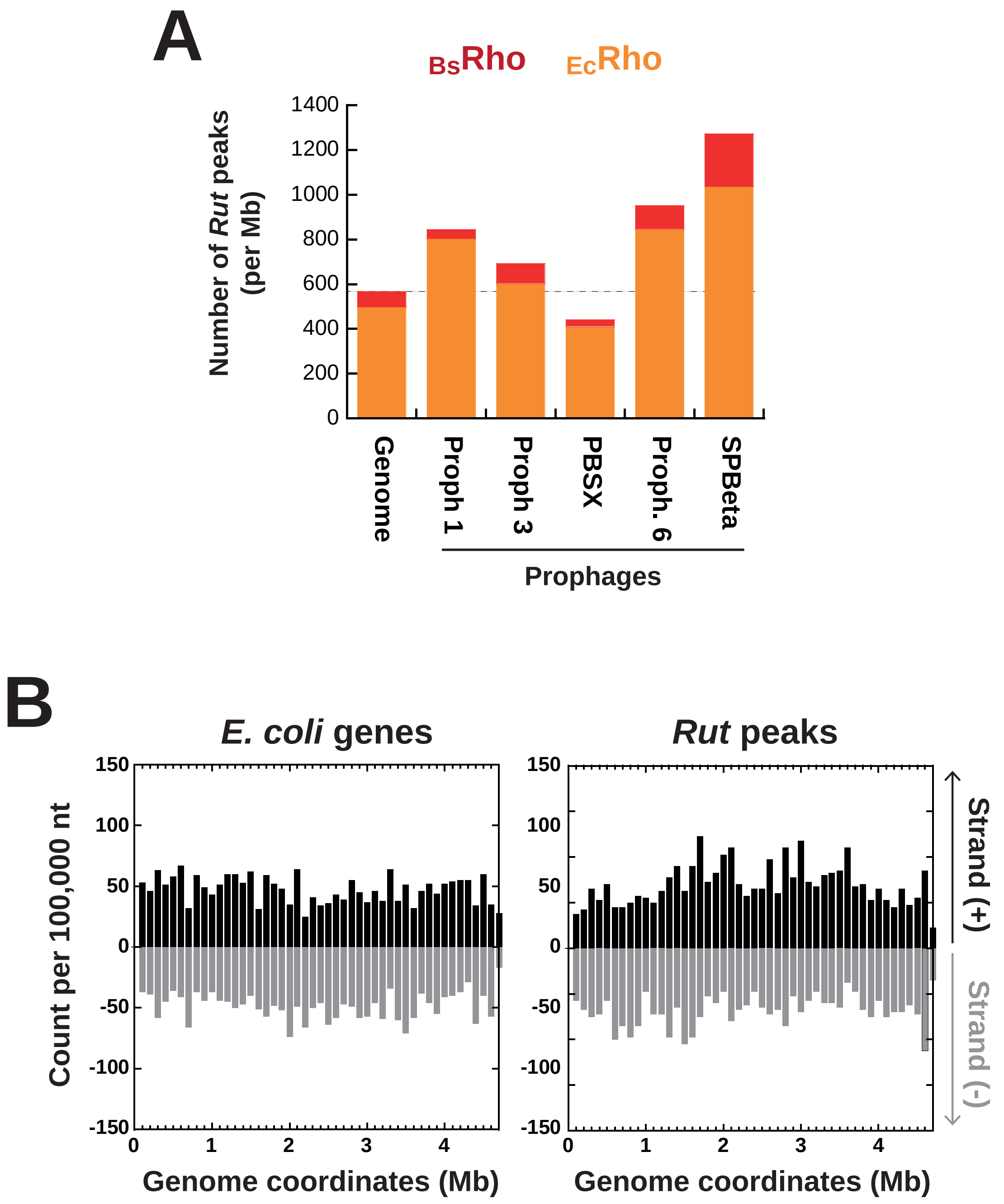

Supplementary Figure S7: (A) Richness in *Rut* peaks in prophages as compared to the whole *B. subtilis* genome. **(B)** distribution of genes and *Rut* peaks along the *E. coli* MG1655 genome. *Rut* peak data were taken from Delaleau et al., 2022 (-NusG condition).

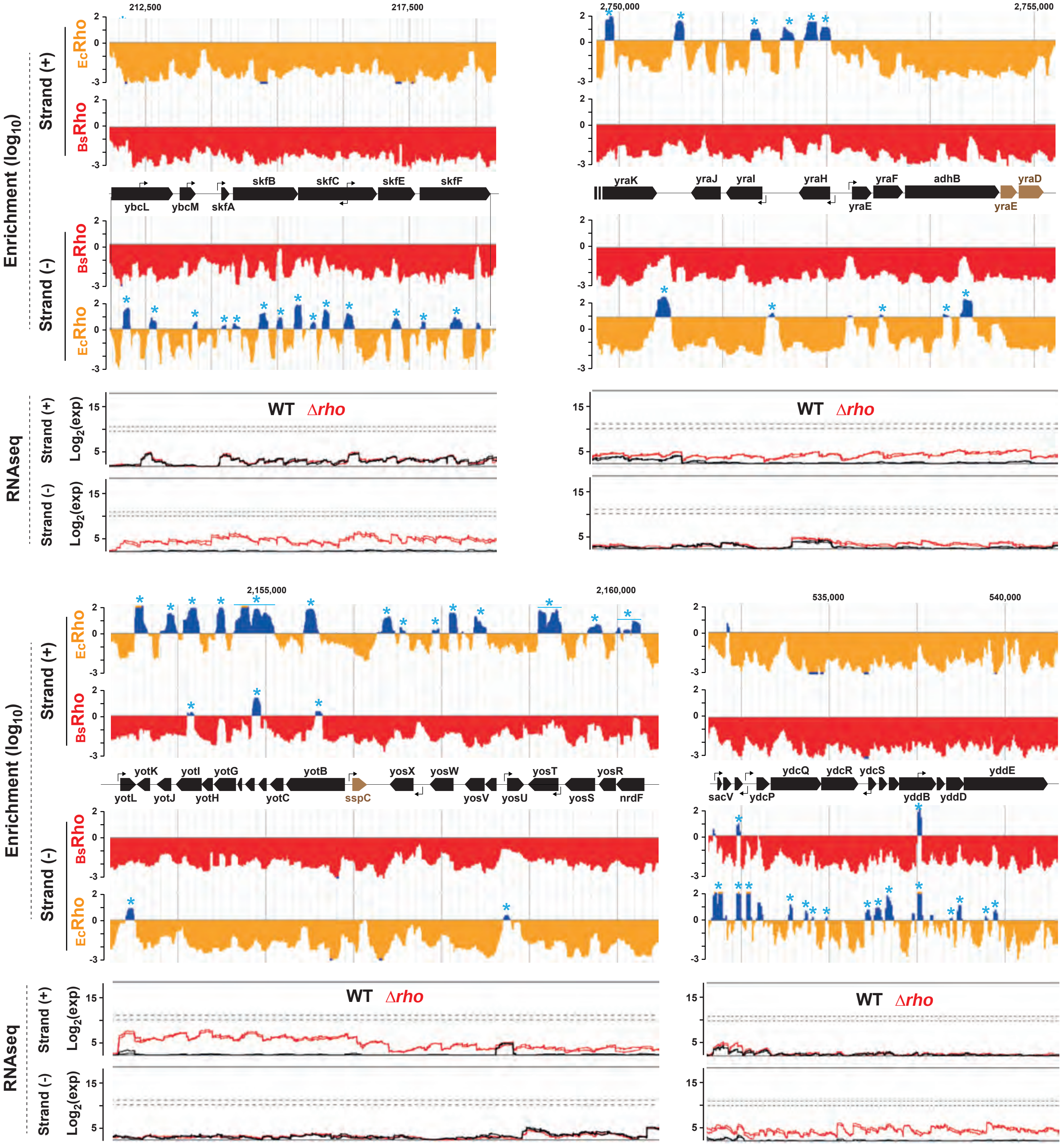

Supplementary Figure S8: Examples of H-SELEX enrichment profiles (Log_{10} scale) for regions of the *B. subtilis* genome that are particularly enriched in *Rut* peaks in antisense orientation (different scales along x-axis). Positive values are in dark blue while negative values are in orange ($_{Ec}$Rho) or red ($_{Bs}$Rho). Validated *Rut* peaks are marked by cyan asterisks. Genoscapist snapshots of RNAseq profiles for the WT and Δ*rho* strains (Bidnenko et al., 2023) are shown for comparison. Sporulation genes are in brown.

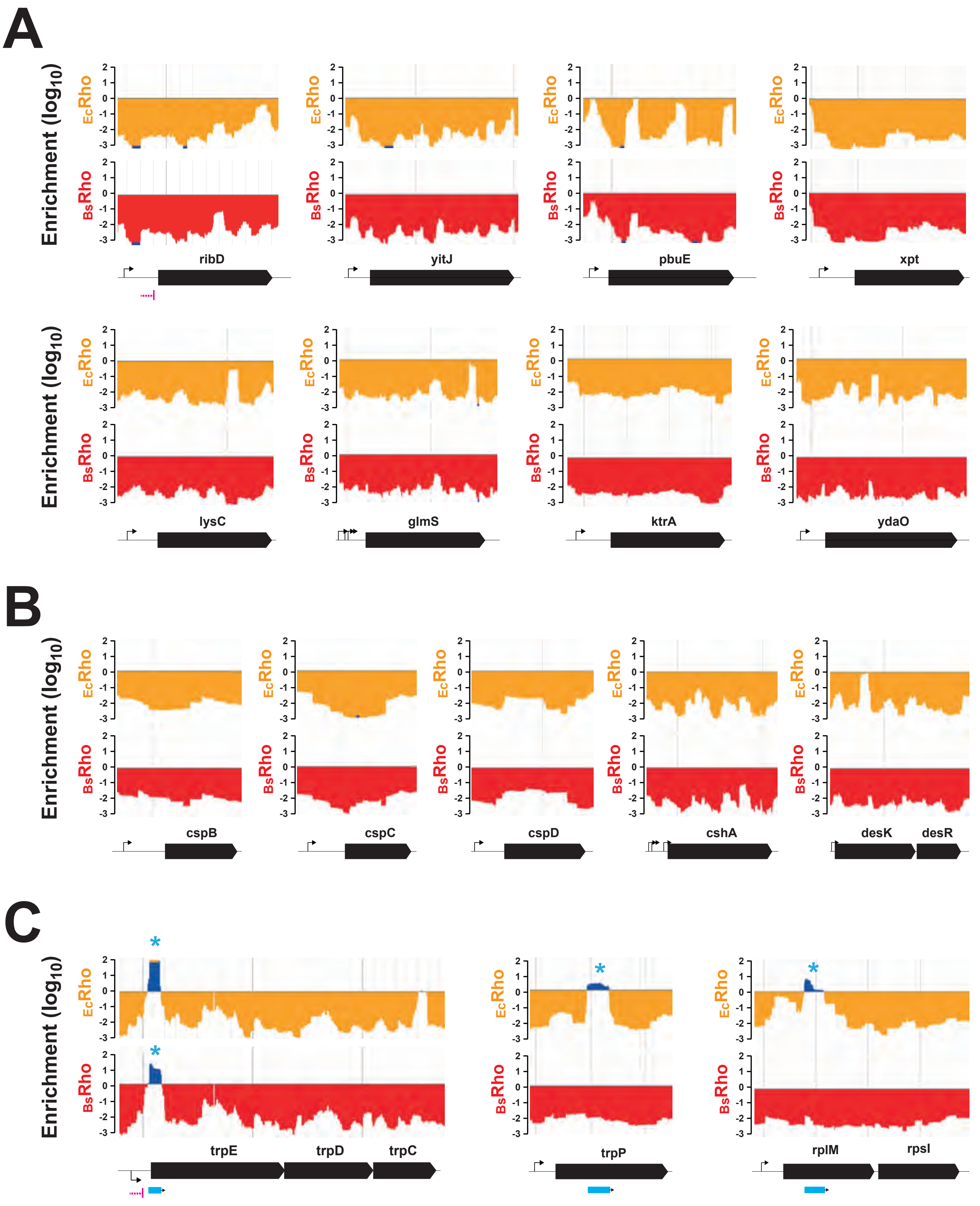

Supplementary Figure S9: Examples of enrichment profiles (Log$_{10}$ scale) for **(A)** genes regulated by a metabolite-dependent riboswitch, **(B)** cold shock genes and **(C)** genes regulated by a protein-dependent riboswitch (different scales along x-axis). Positive values are in dark blue while negative values are in orange ($_{Ec}$Rho) or red ($_{Bs}$Rho). Validated *Rut* peaks are marked by cyan asterisks and rectangles. Pink symbols mark *in vivo* transcript 3'-ends assigned to either Rho-stimulated IT (*ribD*) or RDTT (*trpE*) (Mandell et al., 2022). In the case of *ribD*, the closest *Rut* peak is located within the downstream *ribA* gene (see Table S2). For simplicity, antisense profiles are not shown.

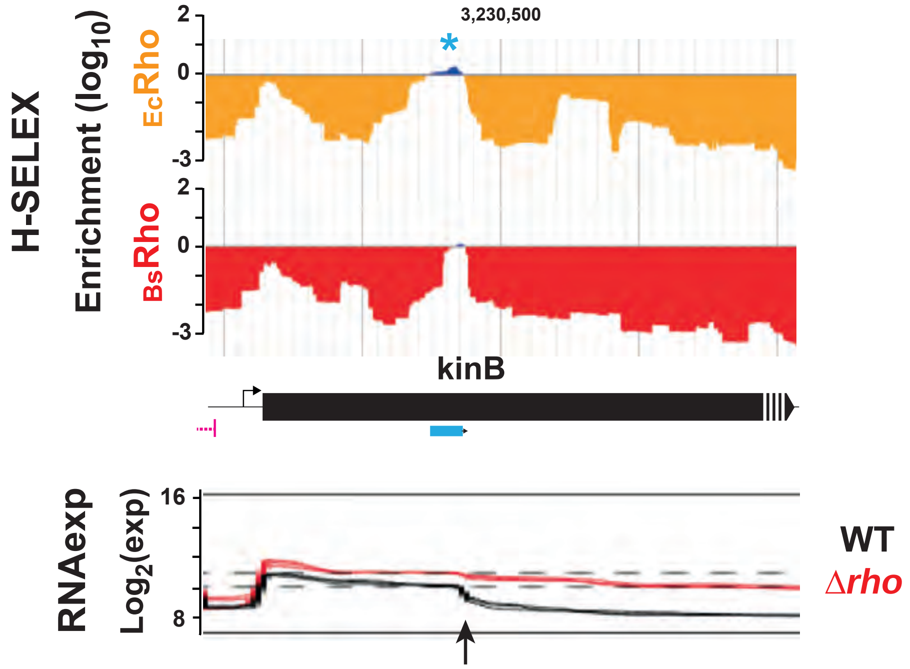

Supplementary Figure S10: H-SELEX enrichment profile (Log_{10} scale) for the *kinB* gene discussed in the main text. Positive values are in dark blue while negative values are in orange ($_{Ec}$Rho) or red ($_{Bs}$Rho). The validated *Rut* peak is marked by cyan asterisk and rectangle. A Genoscapist snapshot of the microarray transcription profiles (RNAexp) (Nicolas et al., 2012) shows a marked downshift in WT transcription in the upstream region of *kinB* (black arrow).

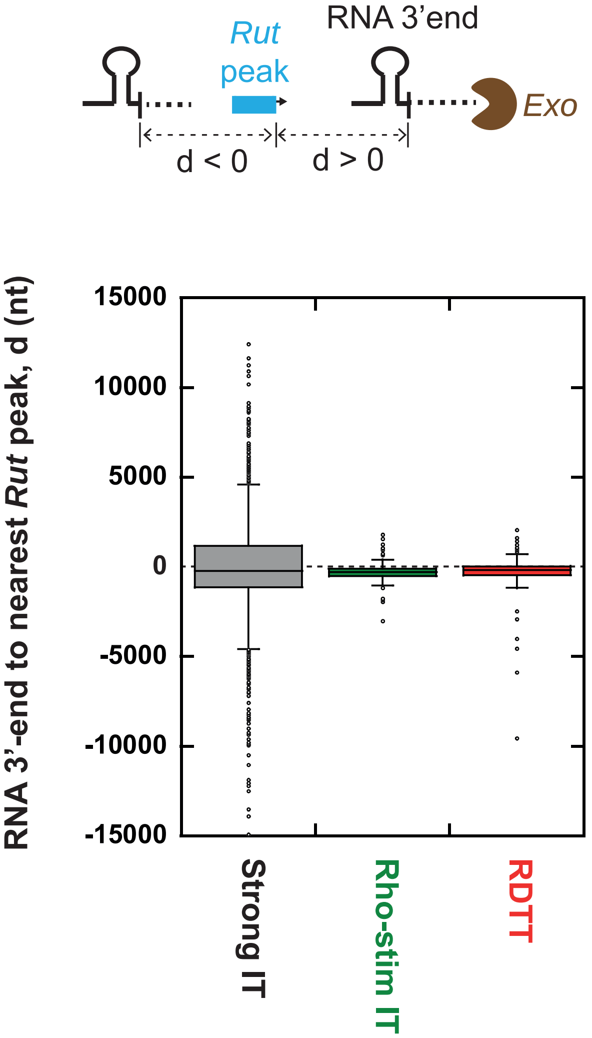

Supplementary Figure S11: Comparison of the *Rut* peak dataset (this work) with theTerm-seq dataset from Mandell et al., 2022. The boxplots shown here and the probability plot of Figure 4E are two different representations of the same data, provided to facilitate the comparison of the datasets.
